# [1, 8]-Naphthyridine derivatives as dual inhibitor of alkaline phosphatase and carbonic anhydrase

**DOI:** 10.1186/s13065-023-01052-8

**Published:** 2023-10-25

**Authors:** Salman Alrokayan, Tajamul Hussain, Salman Alamery, Arif Ahmed Mohammed, Abid Mahmood, Syeda Abida Ejaz, Peter Langer, Jamshed Iqbal

**Affiliations:** 1 Research Chair for Biomedical Application of Nanomaterials, Biochemistry Department, College of Science, King Saud University, 11451 Riyadh, Saudi Arabia; 2 Biochemistry Department, College of Science, King Saud University, 11451 Riyadh, Saudi Arabia; 3 Centre of Excellence in Biotechnology Research, King Saud University, 11451 Riyadh, Saudi Arabia; 4 Centre for Advanced Drug Research, COMSATS University Islamabad, Abbottabad Campus, Abbottabad, 22060 Pakistan; 5 Department of Pharmaceutical Chemistry, Faculty of Pharmacv, The Islamia University of Bahawalpur, Bahawalpur, 63100 Pakistan; 6 Institut Für Chemie, Universität Rostock, A.-Einstein-Str. 3a, 18059 Rostock, Germany

**Keywords:** Carbonic anhydrase, Alkaline phosphatase, [1, 8]-Naphthyridine derivatives, Simulation studies, DFT studies

## Abstract

[1,8]-Naphthyridine derivatives have been reported to possess important biological activities and may serve as attractive pharmacophores in the drug discovery process. [1,8]-Naphthyridine derivatives **(1a-1l)** were evaluated for inhibitory potential for isozymes of carbonic anhydrase (CA) and alkaline phosphatase (ALP). CAs have been reported to carry out reversible hydration of CO_2_ into HCO_3_^−^, secretion of electrolytes, acid–base regulation, bone resorption, calcification, and biosynthetic reactions. Whereas ALPs hydrolyze monophosphate esters with the release of inorganic phosphate and play an important role in bone mineralization. Both enzymes have been found to be over-expressed and raised functional activities in patients suffering from rheumatoid arthritis. The discovery of dual inhibitors of these enzymes may provide a synergistic effect to cure bone disorders such as rheumatoid arthritis and ankylosing spondylitis. Among the test compounds, the most potent inhibitors for CA-II, CA-IX, and CA-XII were **1e**, **1g,** and **1a** with IC_50_ values of 0.44 ± 0.19, 0.11 ± 0.03 and 0.32 ± 0.07 µM, respectively. [1,8]-Naphthyridine derivatives **(1a**–**1l)** were approximately 4 folds more potent than standard CA inhibitor acetazolamide. While in the case of ALPs, the most potent compounds for *b*-TNAP and *c*-IAP were **1b** and **1e** with IC_50_ values of 0.122 ± 0.06 and 0.107 ± 0.02 µM, respectively. Thus, synthesized derivatives proved to be 100 to 800 times more potent as compared to standard inhibitors of *b*-TNAP and *c*-IAP (Levamisole and L-phenyl alanine, respectively). In addition, selectivity and dual inhibition of [1,8]-Naphthyridine derivatives confer precedence over known inhibitors. Molecular docking and molecular simulation studies were also conducted in the present studies to define the type of interactions between potential inhibitors and enzyme active sites.

**Supplementary Information:**

The online version contains supplementary material available at 10.1186/s13065-023-01052-8.

## Introduction

Carbonic anhydrases (CAs; EC: 4.2.1.1) are a family of pervasive Zn^2+^ metalloenzymes found in almost all living organisms, from prokaryotic cells to more complex eukaryotes. CAs catalyze the reaction of reversible hydration of CO_2_ via two-step reactions to yield HCO_3_^−^ and a proton [1]. To date, 16 distinct mammalian CA isozymes having specific activity, physiological roles, kinetic properties, tissue specificity, sub-cellular localization, and susceptibility to various inhibitors have been identified. Carbonic anhydrase I, II, III, VII, and XIII are cytosolic isozymes; CA-IV, IX, XII, and XIV are trans-membrane; CA-VA, and VB are present in mitochondrial.CA-VI is a secretory protein commonly found in saliva and breast milk [2]. Cell surface CA-IX and XII isoforms have high expression in cancerous cells and are responsible for cell adhesion, proliferation, and mutagenesis. CA-IX has minimal expression in normal tissues, whereas it was found to be over-expressed on the cell surface of solid tumors. High expression of CA-IX and CA-XII in hypoxic tumors, coupled with the poor prognosis and the aggressive phenotype, make them candidate targets for cancer therapy [3].

Alkaline phosphatases (APs, EC: 3.1.3.1), belong to the family of ecto-nucleotidases, are metalloenzymes having two Zn^2+^ and one Mg^2+^ ions present in active sites for optimal activities of enzymes [4]. Alkaline phosphatases are classified into two categories; tissue-specific APs and tissue non-specific alkaline phosphatase (TNAP). Tissue-specific APs are further subdivided into placental (PLAP), intestinal (IAP), and germ cell (GCAP) [5]. APs catalyze nucleotides and release inorganic phosphate (Pi). APs can hydrolyze a range of substrates, i.e., glucose-phosphates, phosphatides, and inorganic phosphates [6]. APs are assumed to be imperative in differentiating osteoblasts adipocytes and produce adenosine by sequential de-phosphorylation [6–8]. TNAP is predominantly found in bone and other mineralizing tissues as it regulates mineralization and bone formation [9]. Decreased levels of TNAP lead to skeletal hypo-mineralization [10]. Overexpression of TNAP causes mineral deposition in soft tissues and also causes osteoarthritis [11]. IAP regulates the accumulation of lipids and is involved in adipogenesis. Moreover, it protects against bacterial lipopolysaccharides (LPS) [12]. In the duodenum, the gastric proton is neutralized with secreted bicarbonate to generate carbon dioxide and water, whereas IAP and cytosolic CAs are responsible for maintaining homeostasis [13]. Selective inhibition of IAP inhibits bacterial growth [14].

**Fig. 1:**
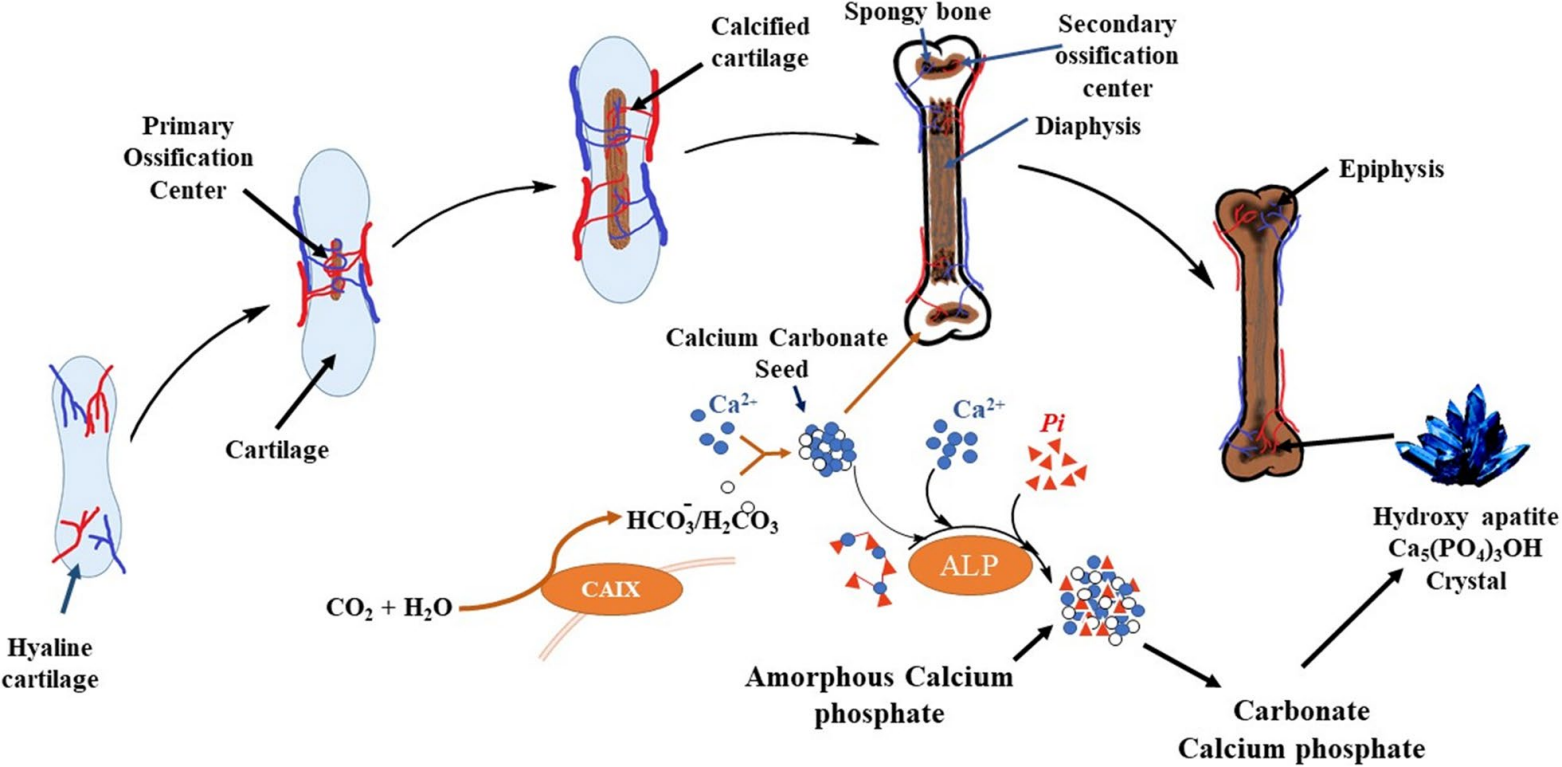
Mineral deposition by two enzymes. **a** Carbonic anhydrase (CA) and **a** alkaline phosphatase (ALP), has been reported to be done in three steps. Step 1: CA catalyzes the reaction that leads to the formation of calcium carbonate bio-seed. Step 2: Non-enzymatic reaction, that transforms carbonate-phosphate by using the orthophosphate generated as a result of ALP-mediated hydrolysis of polyphosphate. Step 3: Formation of amorphous calcium carbonate as well as hydroxyapatite crystals [15, 21]

Alkaline phosphatase and carbonic anhydrases are involved in mineral deposition in bone which may lead to rheumatoid arthritis [15]. It has been reported that CA-IX and CA-XII over-express in the inflamed synovium of patients having juvenile idiopathic arthritis [16, 17]. The raised activity of serum alkaline phosphatase has also been observed in patients affected with rheumatoid arthritis [18, 19]. Inhibition of both enzymes may have a synergistic effect in treating this pathological condition. Designing and synthesis of compounds that inhibit both enzymes can be very fruitful for treating arthritis [20]. Figure 1 shows that CA and ALP are involved in bone mineralization.

Naphthyridine derivatives are reported to exhibit antimicrobial, antiviral, anti-inflammatory, immune-modulatory, and anti-cancerous activities [22, 23]. Nalidixic acid was reported as the first antimicrobial agent in 1962. According to the literature [1, 8]-Naphthyridine derivatives are potential antihypertensive molecules. Some heterocyclic condensed [1, 8]-Naphthyridine derivatives were also reported as antimicrobial agents [24, 25]. In short, the spectrum of activities of these derivatives includes antiviral, anticancer, and anti-inflammatory activities [25, 26]. These derivatives are also reported to show immune-modulatory and anti-cancerous activity [27]. In the current studies, the role of [1, 8]-Naphthyridine derivatives was investigated for inhibitory potential against CA-II, IX, XII, and also for *b*-TNAP and *c*-IAP. The experimental results were subjected to molecular docking and MD simulations to study the possible interactions among active site amino acids and inhibitors.

## Results and discussion

### Chemistry of [1, 8]-Naphthyridine derivatives

About 12 derivatives of [1, 8]-Naphthyridine (1a–1l) were synthesized by Lewis acid-mediated cycloisomerization of ortho-alkynyl-*N*-pyrrolylpyridines. This methodology was found to be operationally simple, versatile, and highly efficient. This synthesis scheme allows the introduction of different functional groups without the need for any specific catalyst or other additives. Sonogashira coupling reaction was used to synthesize the starting material (3-alkynyl-2-([1H]-pyrrol-1-yl)pyridines) of the chemical reaction. Under the best-optimized conditions, triethylamine (3 eq.) as a base and PdCl_2_ (0.03 eq.) as a catalyst were used in acetonitrile at 50 °C for 6 h. This optimized reaction gave the desired products an efficient yield of about 88%. In the final step of the synthesis scheme, cyclization was 3-alkynyl-2-([1H]-pyrrol-1-yl)pyridines was done in the presence of xylene and PtCl_2_ as a catalyst to synthesize [1, 8]-Naphthyridine derivatives (1a-1l). For the structural elucidation of these compounds, various spectroscopic techniques were used, including ^1^HNMR, ^13^C NMR, FT-IR, MS, and HRMS [28]. These synthesized series of [1, 8]-Naphthyridine derivatives were further screened for enzymatic activities.

## Structure–activity relationship (SAR) of [1, 8]-Naphthyridine derivatives for ***h***-CAII, CAIX, CAXII, ***b-***TNAP*** and c-***IAP

The synthesized [1, 8]-Napthyridine derivatives were analyzed for inhibitory potential towards carbonic anhydrase II, IX, and XII. Clinically used acetazolamide (AZM) was used as a positive inhibitor of CA. All compounds had excellent inhibition activity in low micromolar concentrations (Table 1).

**Table 1:** In vitro assay results of compound **1a-l** against *h-*CAII, *h-*CAIX, and *h-*CAXII. Acetazolamide (AZM) was used as a standard inhibitor

Compounds codes	CAII	CAIX	CAXII
	IC_50_ (µM) ± SEM^a^/% inhibition^b^		
1a	1.28 ± 0.16	0.86 ± 0.47	0.32 ± 0.07
1b	1.26 ± 0.44	15.1%	1.51 ± 0.72
1c	0.90 ± 0.5	0.70 ± 0.27	0.96 ± 0.28
1d	17.0%	1.36 ± 0.48	2.4 ± 0.98
1e	0.44 ± 0.19	1.61 ± 0.26	0.51 ± 0.41
1f	11.0%	0.99 ± 0.12	4.3 ± 2.2
1 g	0.10 ± 0.04	0.11 ± 0.03	3.05 ± 0.92
1 h	1.46 ± 0.96	0.74 ± 0.51	0.80 ± 0.69
1i	25.0%	0.87 ± 0.51	0.46 ± 0.12
1j	2.14 ± 0.3	1.65 ± 0.98	0.51 ± 0.26
1 k	1.63 ± 0.55	35.1%	1.80 ± 0.42
1 l	1.99 ± 0.71	3.2 ± 1.52	1.50 ± 0.31
Acetazolamide	1.19 ± 0.42	1.08 ± 0.03	1.55 ± 0.37

^a^n = 3 results were obtained from three individual experiments

^b^Percentage of inhibition measured at 100 µM final concentration of selected compounds

Data obtained from the inhibition of CA-II suggests that one of the most potent compound was **1e** with IC_50_ (µM) ± SEM value of 0.44 ± 0.19. The potency of this series was due to the substitution of R group. The most potent inhibitor of CA-II among the screened compounds, was found to be **1g** that possess a methoxy substitution at meta position of benzene ring, **Ig** has IC_50_ value of 0.10 ± 0.04 µM. In compound **1e** butane was attached at pyrrolo[1, 8]-Naphthyridine ring resulting in maximum inhibition. While in the case of compounds **1d** and **1i** the toluene was present which significantly decreased its inhibitory action with loss of activity, the substitution of anisole in **1f** was also causing a decrease in inhibition as in **1b**. Inhibition of fluorobenzene-substituted compounds was dependent on fluorine. In compound **1c** fluorine at *para*- position was exhibiting more inhibition with IC_50_ ± SEM value of 0.90 ± 0.5 µM, than *ortho-* position in compound **1j** IC_50_ ± SEM value of 2.14 ± 0.3 µM. There was no significant change with the substitution of benzene or *tert*-butylbenzene. Only three compounds **1d, 1f,** and **1i** have shown less than 50% inhibition due to substitution of toluene and methoxy phenol.

While in the case of CA IX, it was found that the compound **1g** was the most potent compound having an IC_50_ (µM) ± SEM value of 0.11 ± 0.03 µM. Compound **1g** exhibited its activity due to the presence of OMe group at meta position of the ring. Further substitution of this R effected the inhibition. The compound **1c** has fluorobenzene with fluorine at position para position. If this fluorine is transferred from para to *ortho* position as in case of compound **1j**, the IC_50_ is elevated to 1.65 ± 0.98 µM. Substitution of anisole with fluorobenzene had not much effect on its inhibition, while substitution of thiophene or tert-butylbenzene at the same position decreased inhibition drastically. Interestingly substituting pyrrolo[1, 8]-Naphthyridine ring with butane in **1e** also showed effective inhibition with an IC_50_(µM) ± SEM value of 1.61 ± 0.26 µM. Only two compounds **1b** and **1k** have shown inhibition of less than 50%.

All compounds were potent inhibitors of CA-XII. The most potent was **1a** as in the case of CA-XII with IC_50_ (µM) ± SEM value of 0.32 ± 0.07 µM. Compound **1i** is inactive against CA-II, while it shows significant activity against the other two isozymes. Similarly, compound **1d** is inactive against CAII while showing marked activity against CA-IX and CA-XII (Table 2).

**Table 2:** In vitro inhibition assay for compounds **1a-l** against *b*-TNAP and *c*-IAP. Levamisole and L-phenyl alanine were used as standard

Compounds codes	*b*-TNAP	*c*-IAP
	IC_50_ (µM) ± SEM^a^ / % Inhibition^b^	
1a	0.38 ± 0.09	0.38 ± 0.15
1b	0.12 ± 0.06	0.90 ± 0.41
1c	1.01 ± 0.31	5.80 ± 1.90
1d	%inh < 30	0.30 ± 0.20
1e	0.32 ± 0.11	0.10 ± 0.02
1f	0.14 ± 0.12	0.97 ± 0.50
1 g	0.63 ± 0.47	0.35 ± 0.21
1 h	33%	37%
1i	%inh < 30	1.03 ± 0.20
1j	%inh < 20	0.46 ± 0.33
1 k	0.36 ± 0.07	0.32 ± 0.11
1 l	0.24 ± 0.06	1.03 ± 0.40
Levamisole	19.21 ± 0.001	–
L-phenyl alanine	–	80.21 ± 0.001

^a^n = 3 results were obtained from three individual experiments

^b^Percentage of inhibition measured at 100 µM final concentration of selected compounds

Compound **1b** was most potent in the case of *b*-TNAP with IC_50_ ± SEM of 0.12 ± 0.06 µM, where Bu is substituted at para position of the ring. **1b** was found to be 160 times more potent as compared to the standard inhibitor of TNAP, Levamisole (IC_50_ = 19.21 ± 0.001 µM). Compound **1f** exhibited a slight increase in IC_50_ because of substitution of methoxy phenyl at same position. IC_50_ was increased to 0.14 ± 0.12 µM. Compounds **1l, 1e** and **1k** showed relatively close IC_50_ values of 0.24 ± 0.06, 0.32 ± 0.11, and 0.36 ± 0.07 µM, respectively. IC_50_ value of compound **1k** is due to the substitution of the furan ring. In compound **1e** free pentane is substituted at pyrrolo[1, 8]-Naphthyridine ring which is responsible for its activity. In compound **1l** activity is attributed to cyclohexane substituted at pyrrolo[1, 8]-Naphthyridine ring. Substitution with fluorine as in the case of **1j**, toluene in **1i** and anisole in **1h** resulted in loss of activity.

Compound **1e** expressed the highest potency against *c*-IAP as butane is fused with a naphthyridine ring with an IC_50_ xvalue of 0.10 ± 0.02 µM and found to be 800 times more potent as compared to L-phenyl alanine, a standard inhibitor for IAP. Any other substitution resulted in the elevation of IC_50_ value as shown in compound **1c** substitution of fluorine at benzene ring increased IC_50_ value to 5.80 ± 1.90 µM. Substitution of anisole resulted in a loss of activity in **1h**. In compound **1h** anisole is substituted which resulted in a complete loss of activity. Activity in compound **1d** is due to the substitution of toluene at pyrrolo[1, 8]-Naphthyridine ring, having an IC_50_ of 0.30 ± 0.20 µM. Compound **1g** exhibited an IC_50_ value of 0.35 ± 0.21 µM due to substitution of methoxy phenyl at benzene ring attached to pyrrolo[1, 8]-Naphthyridine nucleus.

### Density functional theory (DFT) calculations

**Fig. 2:**
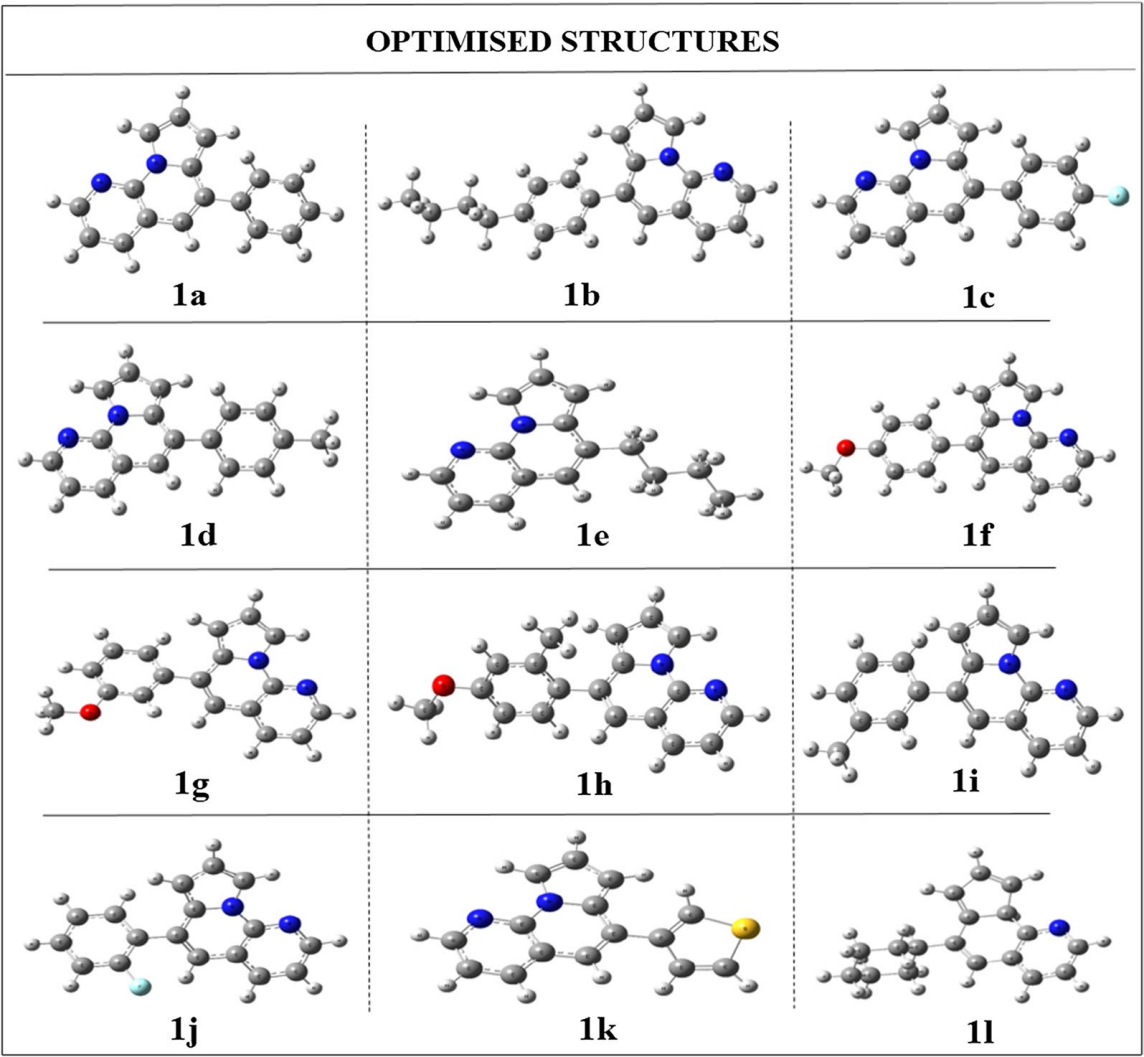
Optimized structures of all [1, 8]-Naphthyridine derivatives (1a–1l) using B3LYP/6-31G level of theory. Blue, yellow and red atoms indicate N, S and O atoms

For the last 30 years, among most popular techniques for doing ab initio calculations on the structure of atoms, molecules, crystals, surfaces, and their interactions is density functional theory (DFT). These calculations are used to understand the electronic behavior of a compound which helps in predicting the chemical reactivity. All the DFT calculations of this study [1, 8]-Naphthyridine derivatives (**1a–1l**) were performed using B3LYP functional theory and 6-31G basis set. The 6-31G is a medium basis set most commonly used for compounds with atoms up to Argon [41]. Using Gaussian software, a number of parameters, including the dipole moment, molecular polarizability, and optimization energy, were determined for all the derivatives [42]. The optimization energy of a compound is the amount of energy required to get the most stable configuration, i.e. lower the optimization energy of a compound higher its stability, which may be represented by its optimized structures. The optimized structures of all the derivatives are given in Fig. 2.

### HOMO and LUMO orbitals

The energy of any compound for its highest occupied and lowest unoccupied molecular orbitals, i.e., HOMO and LUMO, can be used to determine the range of chemical properties related to its electronic distribution [43, 44]. Any molecule with a high HOMO value appears to be an excellent electron donor; electron acceptors have higher LUMO values. Determining local reactivity at various sites in a compound was made more accessible with the help of the overall FMO calculation. A compound's HOMO/LUMO energy difference significantly indicates its reactivity profile. Any compound with a more significant energy gap tends to be less reactive, as indicated by the high hardness value of the compound. According to the DFT results, all 12 compounds were highly reactive, evident from their lowest average energy gap, i.e., ΔEgap = − 0.136, which is almost common for all derivatives. The ΔEgap results for all the other compounds are positive and are given in the Table 3, along with other parameters.

**Table 3:** Optimization energies, HOMO and LUMO energies, and energy gap for compounds **(1a–1l)**

Codes	Optimization energy	Dipole moment	Polarizability (α)	HOMO (eV)	LUMO (eV)	HOMO–LUMO ($\Delta$ eV)
1a	− 764.35	2.26	195.26	− 0.191	− 0.055	0.136
1b	− 921.57	2.49	247.47	− 0.189	− 0.053	0.136
1c	− 863.56	2.38	195.71	− 0.196	− 0.059	0.137
1d	− 803.66	2.55	210.83	− 0.189	− 0.053	0.136
1e	− 690.57	2.50	172.30	− 0.190	− 0.046	0.144
1f	− 878.84	3.68	218.20	− 0.188	− 0.052	0.136
1 g	− 878.83	3.07	214.58	− 0.189	− 0.053	0.136
1 h	− 867.45	1.45	212.4	− 0.178	− 0.051	0.127
1i	− 803.66	2.58	208.13	− 0.190	− 0.053	0.137
1j	− 863.56	2.14	195.04	− 0.192	− 0.058	0.134
1 k	− 1085.09	2.52	190.95	− 0.193	− 0.058	0.135
1 l	− 751.89	2.23	192.45	− 0.191	− 0.055	0.136

**Fig. 3:**
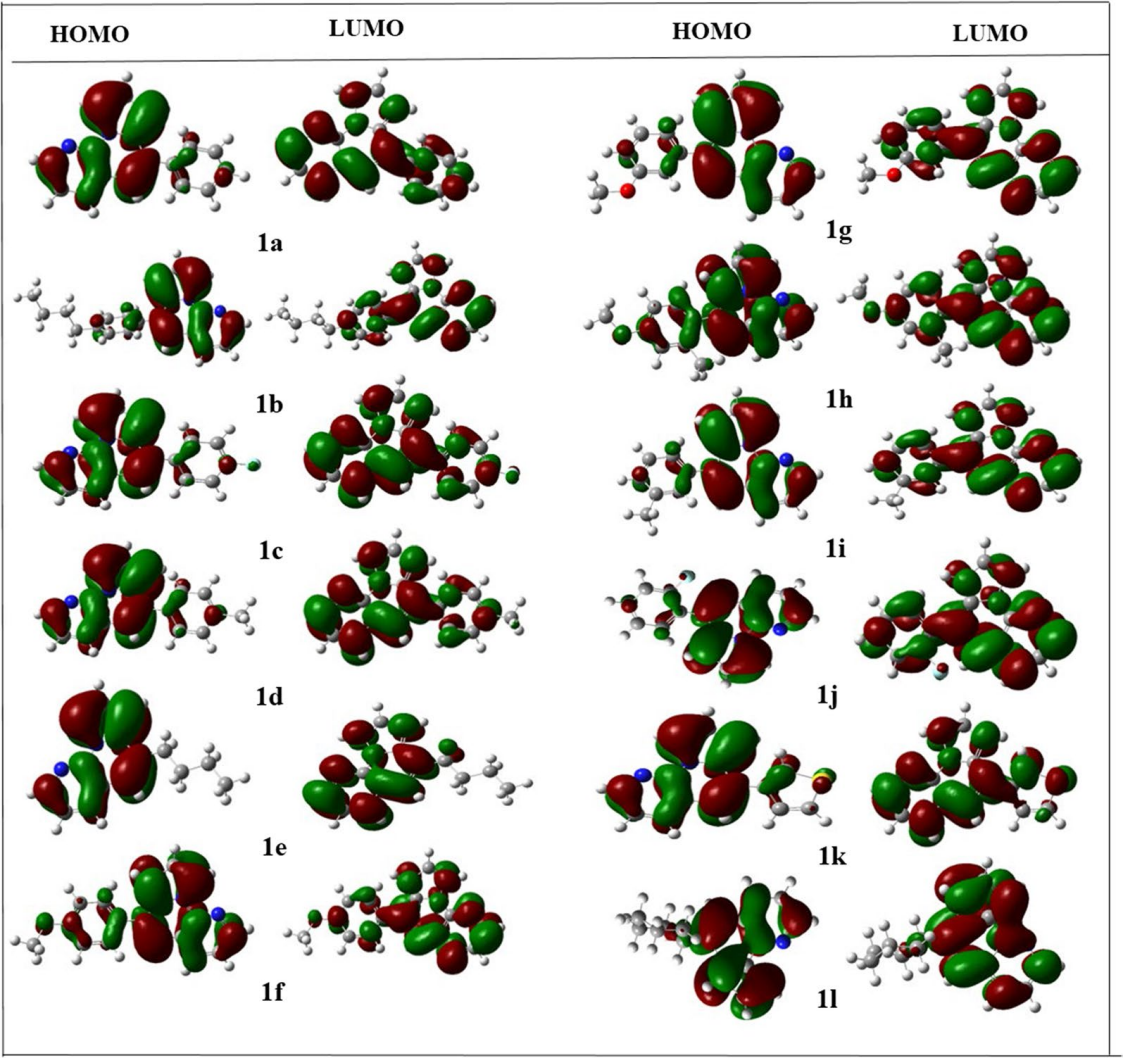
HOMO–LUMO interfacial plots of the orbitals for all compounds. Blue, yellow and red atoms indicate N, S and O atoms. Deep-green and deep-red parts represents the different phases of molecular wave functions

The dipole moment of any substance is related to the charge segregation in a molecule and helps to find its active sites. The higher value of dipole moment speaks for its more polar character with maximum reactive points. According to the DFT results, the derivative **1g** has the highest dipole moment value, i.e., 3.07D, which is consistent with both in vitro and in silico studies. The lowest dipole moment value of **1j** is also relevant to other studies. In the same high polarizability value of compound **1g** augments the DFT findings. The structure of the HOMO and LUMO orbitals for all the compounds are shown in Fig. 3 below, while the polarizability, dipole moment, energy, and relative energy gap are calculated and presented in the Table 3.

### Chemical descriptors

Various parameters used to assess a compound's reactivity include chemical hardness and softness, electrophilicity index, electronegativity, and electronic chemical potential [45]. The chemical formula (ELUMO-EHOMO)/2) can be used to determine the chemical hardness of a chemical system based on its stability and reactivity. The high value of hardness for a compound is related to its least reactivity and high stability. The ability of an atom in a molecule to draw protons towards itself is known as electronegativity, which is quantified by the formula X = -(EHOMO + ELUMO)/2). The electrophilicity index measures a molecule's capacity for accepting electrons by utilizing its electronic chemical potential and chemical hardness. Table 4 contains the values of several chemical descriptors for all substances that were determined to be similar.

**Table 4:** Chemical descriptors for synthesized [1, 8]-Napthyridine derivatives

Codes	Chemical potential µ (eV)	Electronegativity X (eV)	Hardness ƞ (eV)	Softness S (eV−1)	Electrophilicity index ω (eV)
1a	− 0.123	0.123	0.068	7.35	0.111
1b	− 0.121	0.121	0.068	7.35	0.108
1c	− 0.128	0.128	0.069	7.30	0.119
1d	− 0.121	0.121	0.068	7.35	0.108
1e	− 0.118	0.118	0.072	6.94	0.097
1f	− 0.120	0.120	0.068	7.35	0.106
1 g	− 0.121	0.121	0.068	7.35	0.108
1 h	− 0.127	0.127	0.079	7.43	0.123
1i	− 0.122	0.122	0.069	7.30	0.108
1j	− 0.125	0.125	0.067	7.46	0.117
1 k	− 0.126	0.126	0.068	7.41	0.117
1 l	− 0.127	0.127	0.068	7.35	0.111

### Molecular docking studies

**Fig. 4:**
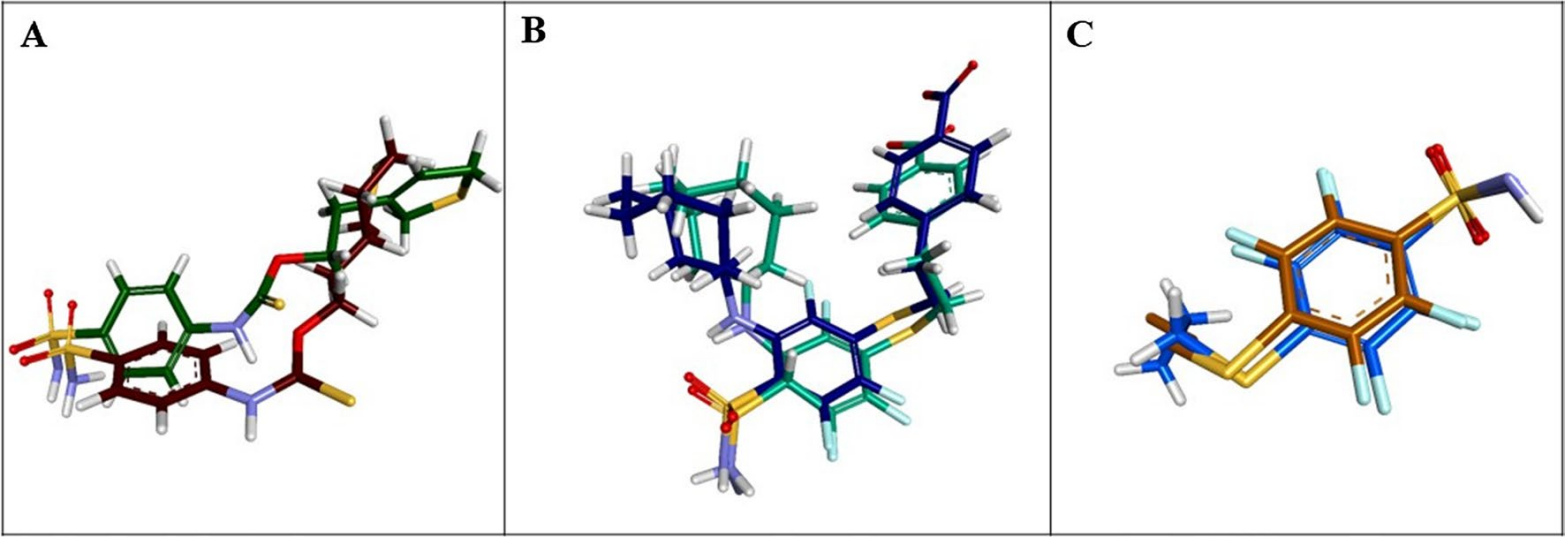
**A** Cognate Ligand of 3K34 with RMSD of 0.7168 Å, **B** Cognate Ligand of 6G9U with RMSD of 0.8243 Å, **C** Cognate Ligand of 5MSA with RMSD of 0.6108 Å

The affiliated ligands for CA-II, CA-IX, and CA-XII were subjected to re-docking for the method validation. These cognate ligands' coming out RMSD values below 1 Å endorsed the docking procedure. The re-docking of the cognate ligand for 3K34 protein revealed RMSD value of 0.7168 Å (Fig. 4A), RMSD value for the cognate ligand of 6G9U exhibited 0.8243 Å (Fig. 4B), and the cognate ligand of 5MSA had the RMSD value of 0.6108 Å (Fig. 4C).

CA-II (3K34) was incorporated with its cognate sulfonamide ligand (4-sulfamoyl-phenyl)-thiocarbamic acid O-(2-thiophen-3-yl-ethyl) ester, interacting with Zn^2+^ metal, Thr199, and Gln92 residues in the active pocket of the enzyme. The crystal structure of CA-IX (6G9U) came along with the sulfonamide ligand 4-[2-[3-(cyclooctylamino)-2,5,6-tris(fluoranyl)-4-sulfamoyl-phenyl]sulfanylethyl]benzoic acid inside the active pocket bonded to Zn^2+^ metal, Arg60, Asn62, Gln92, His119, Thr199, and Thr200 residues of the active pocket. CA-XII (5MSA) exhibited its sulfonamide cognate ligand 2,3,5,6-tetrafluoro-4-(propylsulfanyl)benzenesulfonamide bound to Zn^2+^ metal, His91, His93, Glu104, His117, Leu197, Thr198, and Thr199 residues.

### Molecular docking studies against carbonic anhydrase II (3K34)

**Fig. 5:**
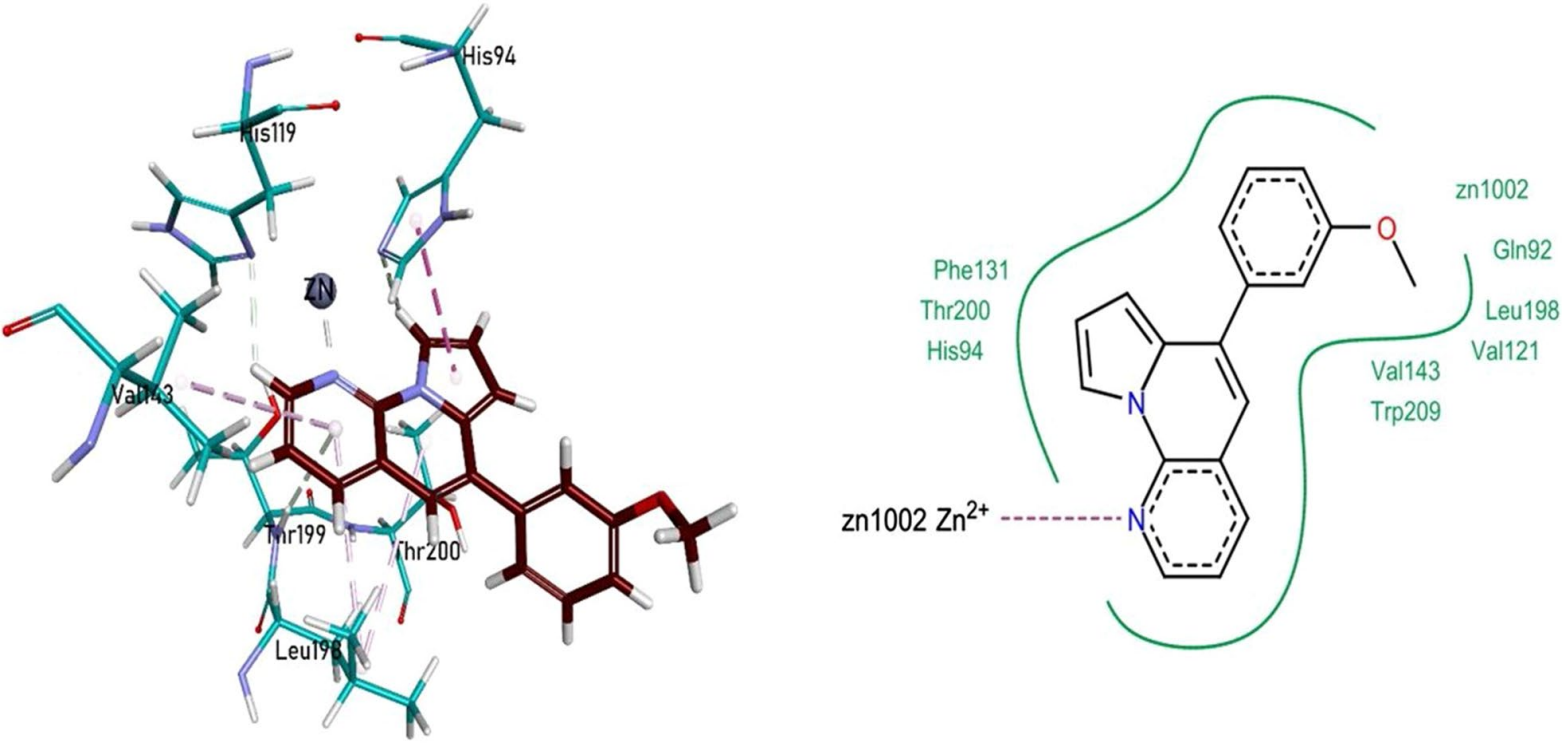
Compound **1g** interactions inside CA II (3k34) active pocket

The in vitro analysis revealed that **1g** was found to be the most potent inhibitor of CA II. The nitrogen heteroatom of pyrrolo-naphthyridine ring of **1g** formed a metallic linkage with Zn^2+^ metal, and the same pyrrolo-naphthyridine ring was involved in hydrogen bonding with His94, His119, and Thr199, while forming π-π linkages with Val143 and Leu198. The compound **1g** also exhibited van der Waals interactions with Gln92, His94, Val121, Phe131, Val143, Leu198, Thr200, and Trp209 residues (Fig. 5).

### Molecular docking studies against carbonic anhydrase IX (6G9U)

**Fig. 6:**
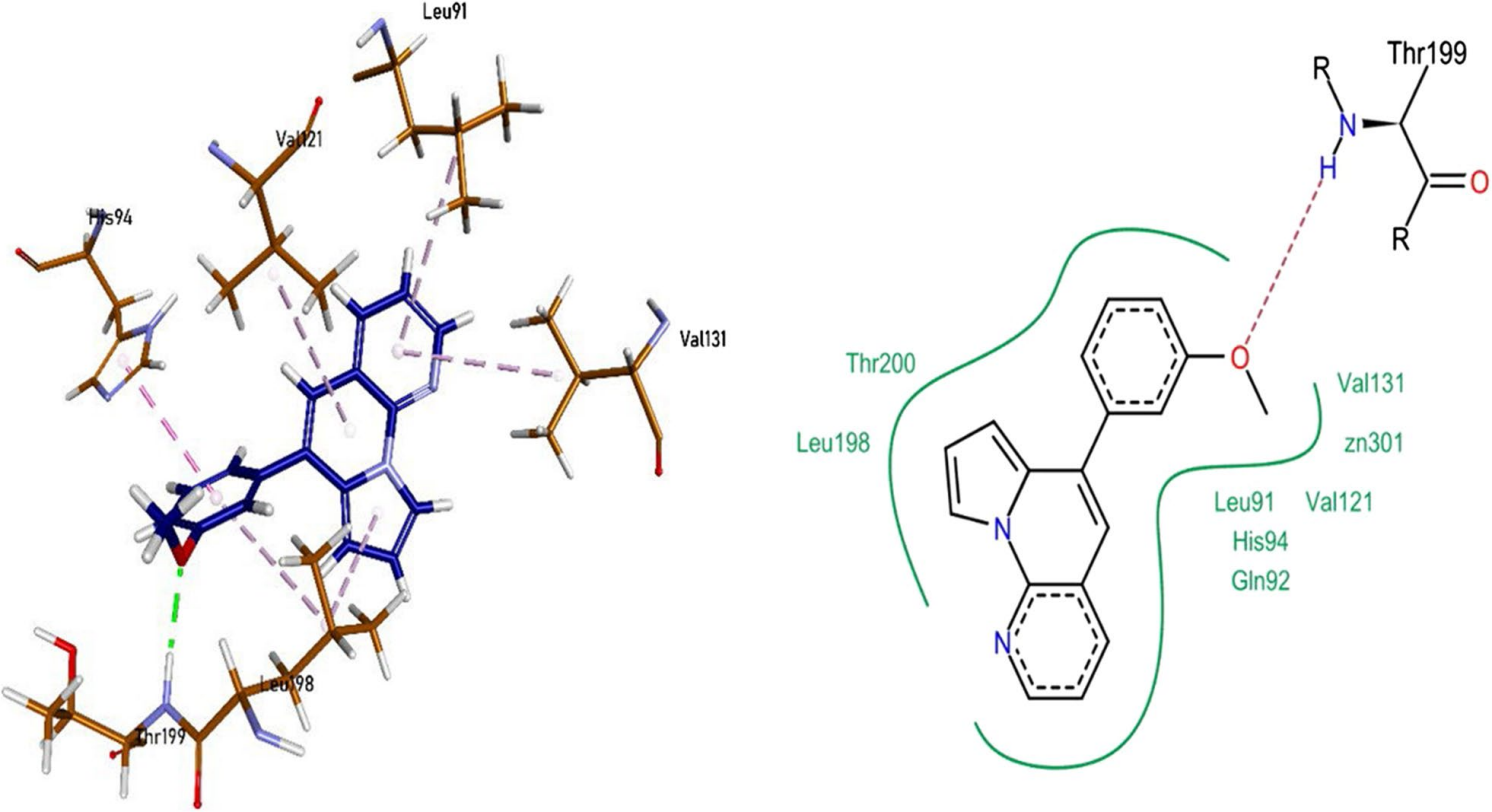
Compound 1g interactions inside CA IX (6G9U) active pocket

The biological assay revealed that 1g also exhibited highest potency towards CA IX. The pyrrolo-naphthyridine ring of **1g** formed π-π linkages with Leu91, Val121, Val131, and Leu198. The methoxyphenyl ring of the compound 1g was involved in π-π interaction with His94 while π-alkyl linkages with Leu198 residue. Thr199 formed a hydrogen bond with the methoxy moiety on the methoxyphenyl ring. The compound **1g** also exhibited van der Waals interactions with Leu91, Gln92, His94, Val121, Val131, Leu198, and Thr200 (Fig. 6).

### Molecular docking studies against carbonic anhydrase-XII (5MSA)

**Fig. 7:**
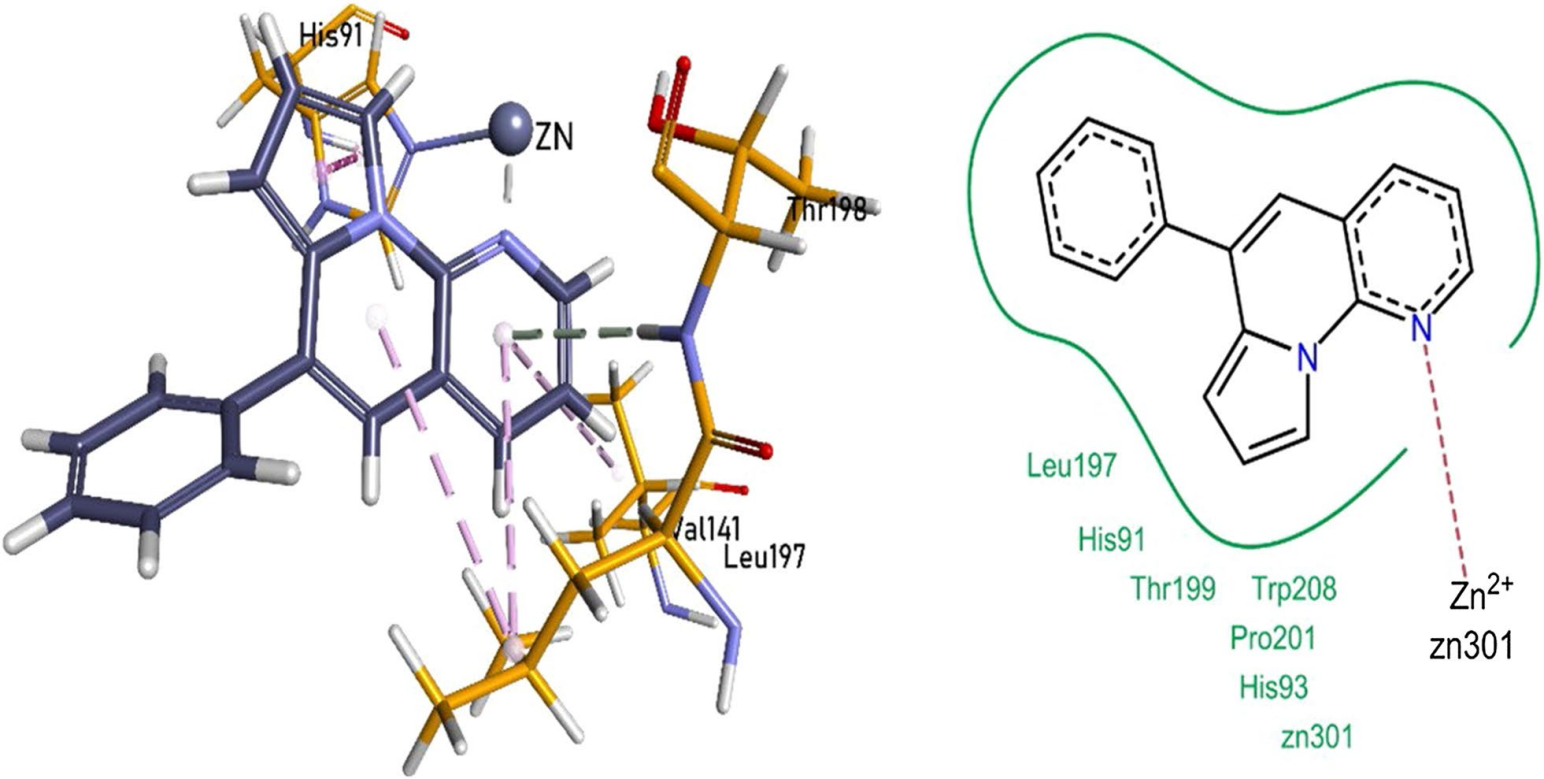
Compound 1a interactions inside CA-XII (5MSA) active pocket

The enzyme inhibition assay revealed **1a and 1i** to be the most potent inhibitors of the carbonic anhydrase XII enzyme. The nitrogen heteroatom of pyrrolo-naphthyridine ring of 1a formed a metallic linkage with Zn^2+^ metal and forming π-π linkages with His91, while π-alkyl bonding with Val141 and Leu197 residues. Pyrrolo-naphthyridine ring established a hydrogen bonding with Thr198 residue. The compound **1a** also exhibited van der Waals interactions with His91, His93, Leu197, Thr199, Pro201 and Trp208 (Fig. 7).

**Fig. 8:**
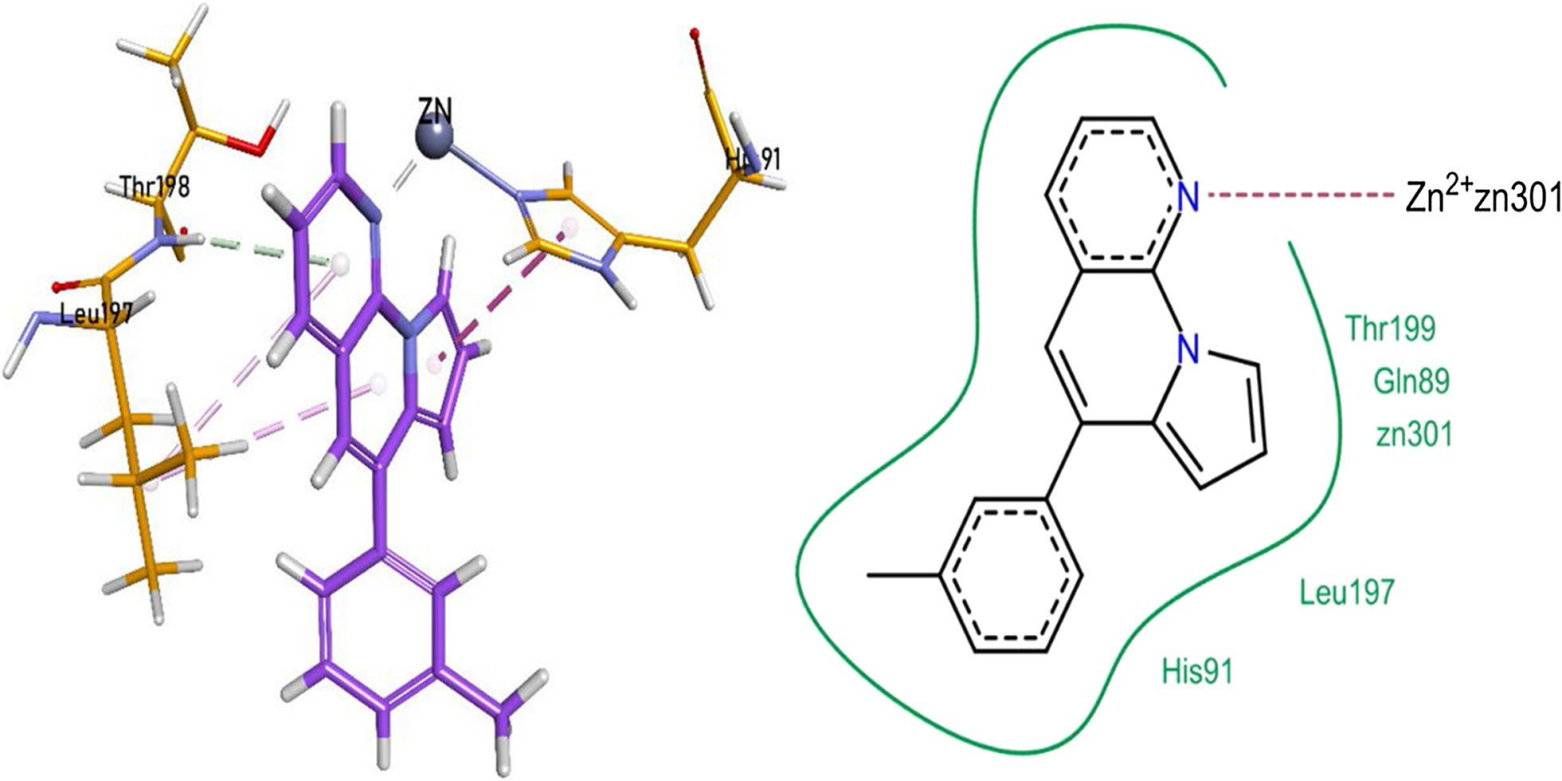
Compound 1i interactions inside CA-XII (5MSA) active pocket

The nitrogen heteroatom of pyrrolo-naphthyridine ring of **1i** formed a metallic linkage with Zn^2+^ metal and forming π-π linkages with His91, while π-alkyl bonding with Leu197 residue. Pyrrolo-naphthyridine ring was also linked to Thr198 through a hydrogen bonding. The compound **1i** also revealed van der Waals interactions with Gln89, His91, Leu197, and Thr199 residues (Fig. 8).

**Fig. 9:**
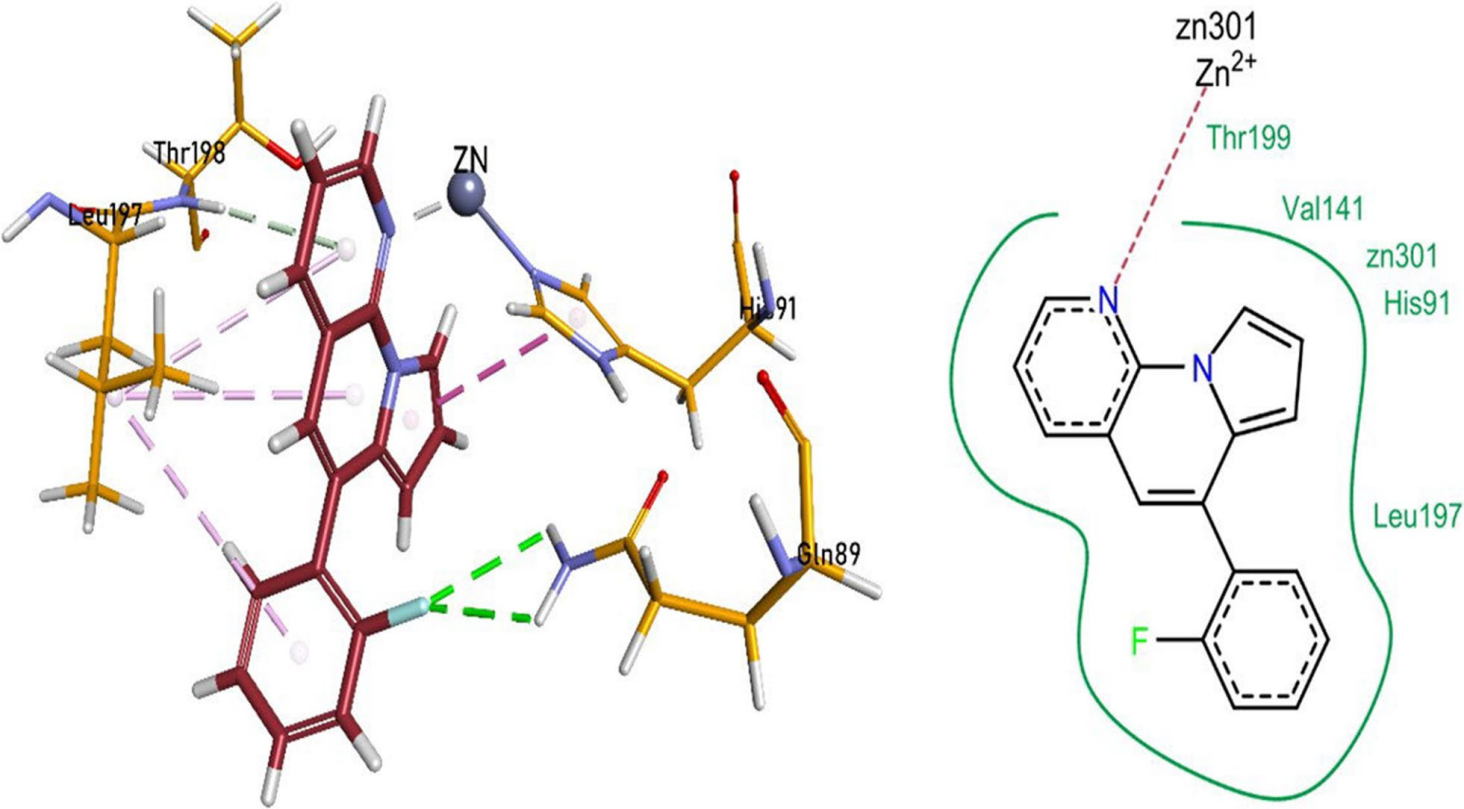
Compound 1j interactions inside CA-XII (5MSA) active pocket

The nitrogen heteroatom of pyrrolo-naphthyridine ring of **1j** formed a metallic linkage with Zn^2+^ metal and forming π-π linkages with His91, while π-alkyl bonding with Leu197 residue. Pyrrolo-naphthyridine ring was also involved in the formation of hydrogen bonding with Thr198 residue. Fluorophenyl moiety of the compound **1j** was involved in the formation of hydrogen bonding with Gln89. The compound **1j** also expressed van der Waals interactions with His91, Val141, Leu197, and Thr199 residues (Fig. 9).

### Molecular docking studies against human intestinal alkaline phosphatase (*h*-IAP)

Initially, the docking methodology was validated by docking the L-phenylalanine as standard inhibitor of IAP into the active pocket of *h*-IAP.

**Fig. 10:**
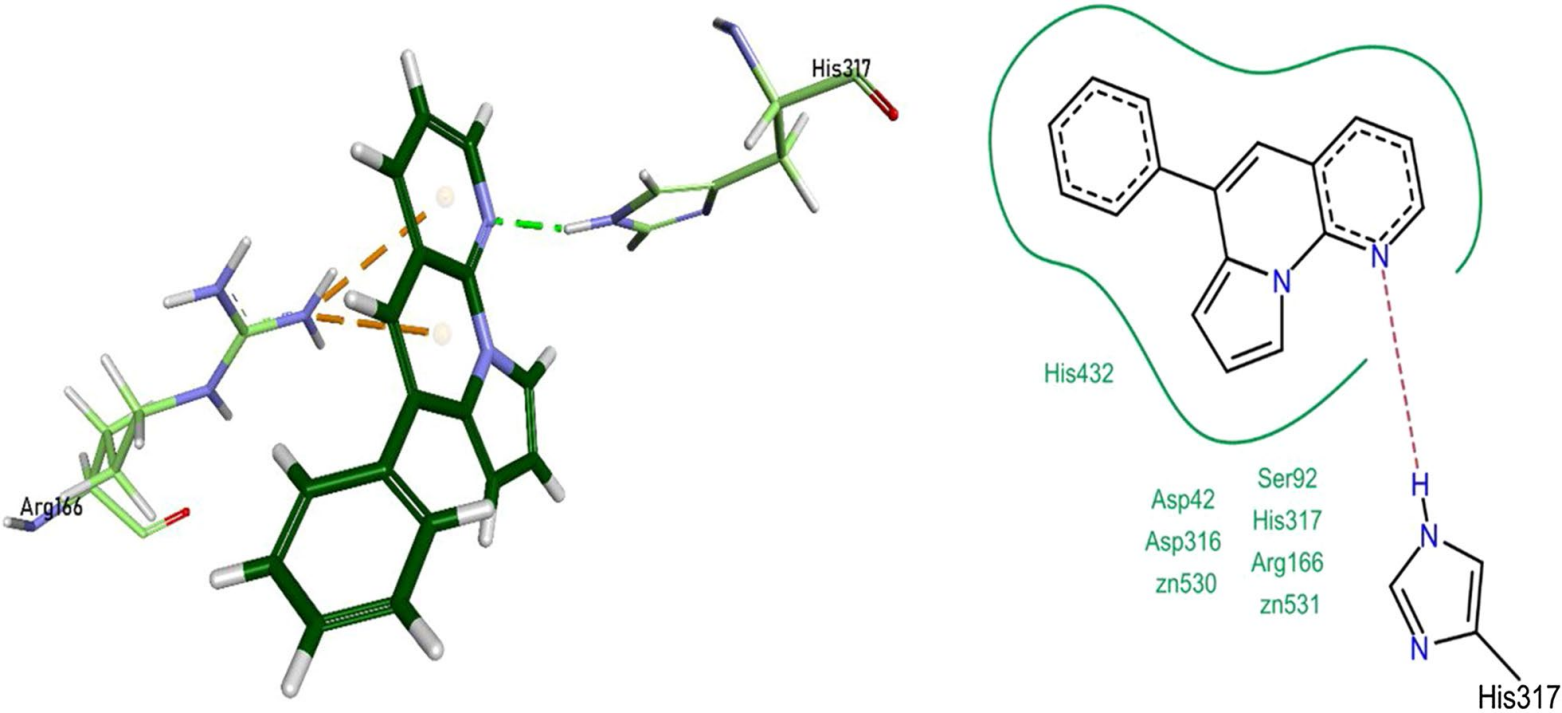
Compound 1a interactions inside h-IAP active pocket

**Fig. 11:**
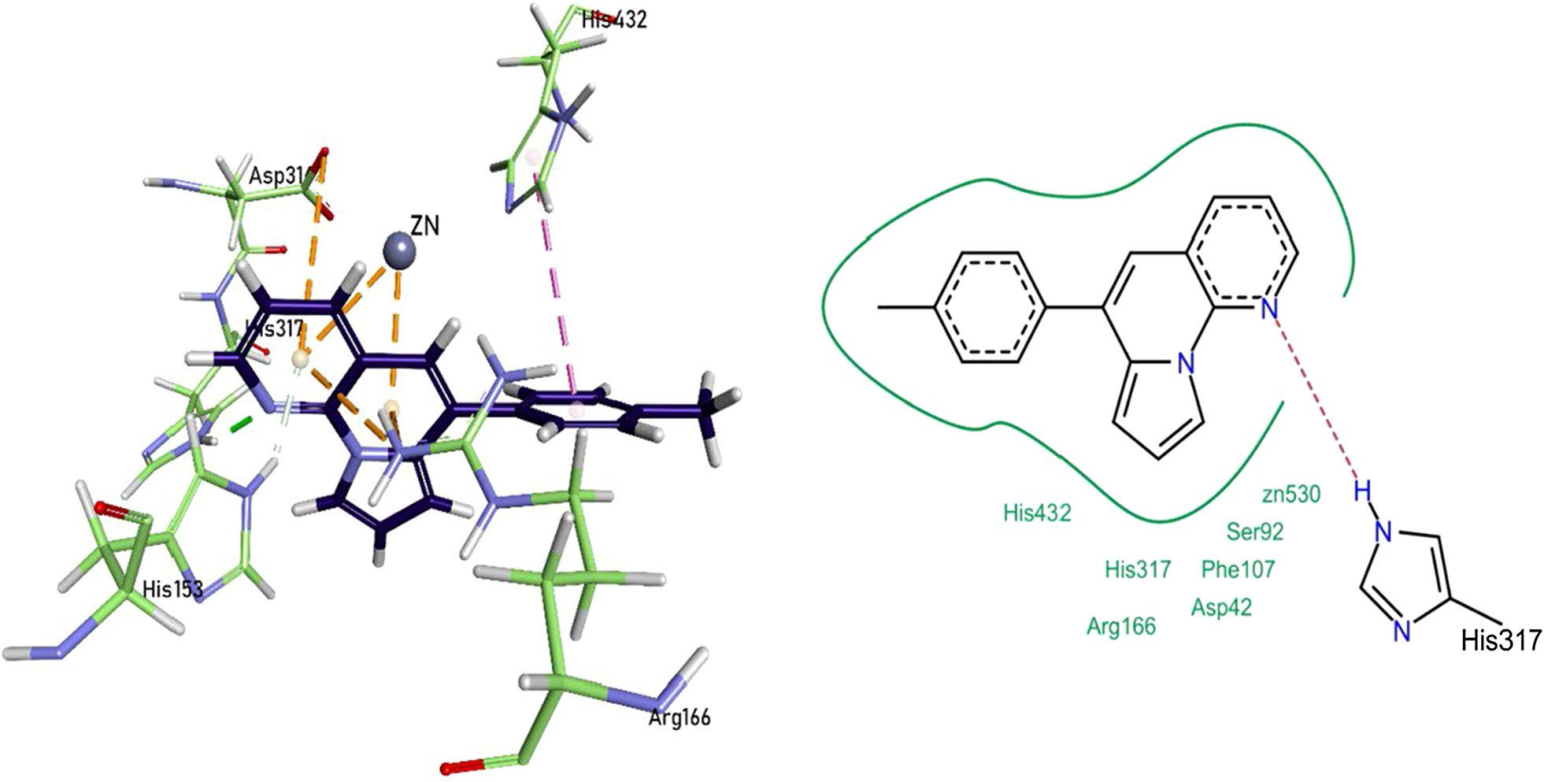
Compound 1d interactions inside h-IAP active pocket

It was observed during the molecular docking that the test compounds imitated the interactions of L-phenylalanine. In vitro analysis found **1a, 1d, 1e**, **1g, 1j,** and **1k** as effective inhibitors of the *h*-IAP protein. The nitrogen heteroatom of pyrrolo-naphthyridine ring of 1a formed a conventional hydrogen bonding with His317 residue, and the pyrrolo-naphthyridine ring itself was involved in the formation π-cationic bonding with Arg166 residue of the protein. The compound **1a** also exhibited van der Waals interactions with Asp42, Ser92, Arg166, Asp316, His317, His437, and the two Zn^2+^ metals found in the active pocket of the enzyme (Fig. 10).

he nitrogen heteroatom of pyrrolo-naphthyridine ring of 1d formed hydrogen bonding with His317 and His153 residues, and the pyrrolo-naphthyridine ring itself was involved in the formation π-cationic bonding with Arg166, Asp316, and Zn^2+^ metal of the active pocket. His432 formed π-π interaction with the phenyl ring of compound **1d**. The compound 1d also exhibited van der Waals interactions with Asp42, Ser92, Phe107, Arg166, His317, and His432 protein residues (Fig. 11).

### Molecular docking studies of test compounds against human tissue non-specific alkaline phosphatase (*h*-TNAP)

The binding residues for test compounds in *h*-TNAP were validated with its known ligand levamisole. It was observed during the molecular docking that the test compounds imitated the interactions of levamisole. In vitro analysis found **1a, 1b, 1e, 1f, 1k,** and **1l** as compelling inhibitors of the *h*-TNAP protein.

**Fig. 12:**
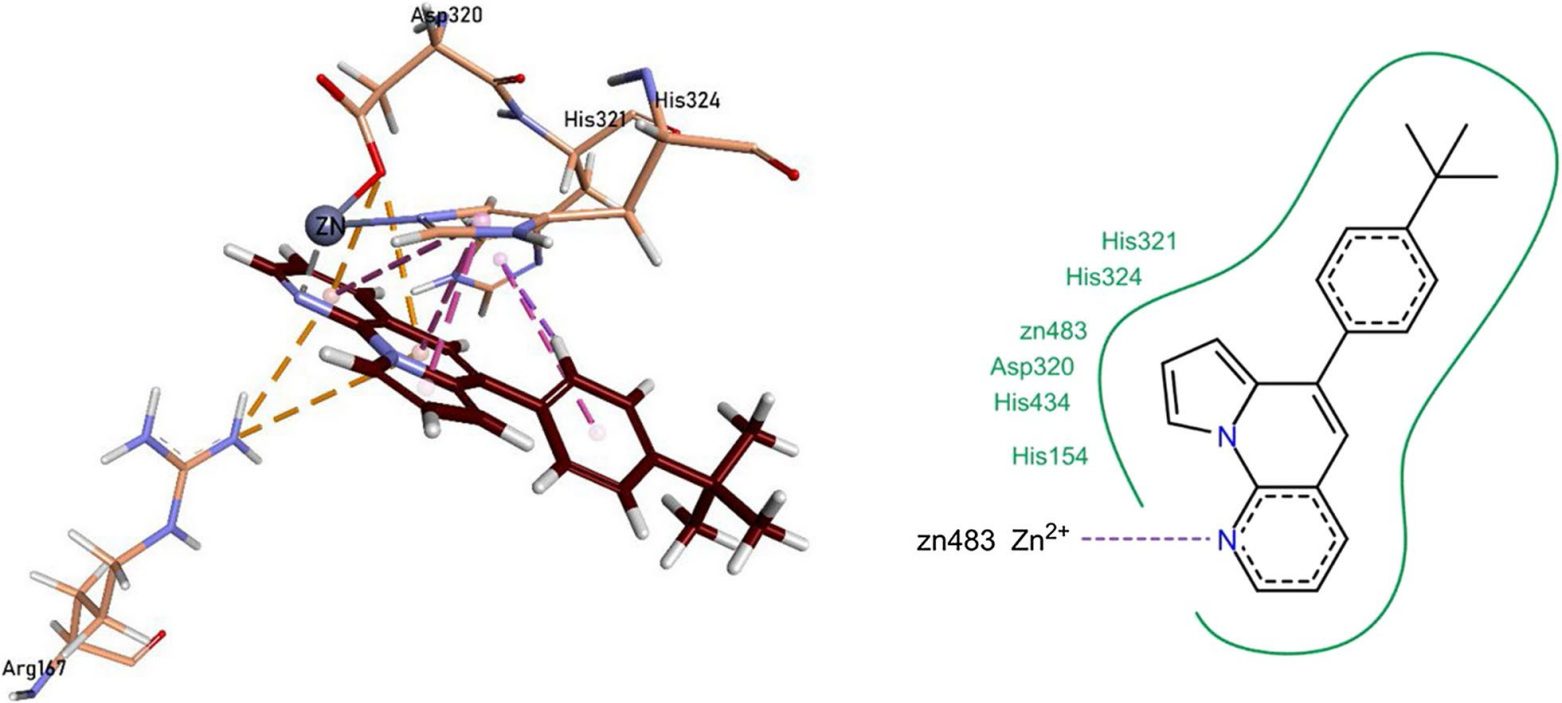
Compound 1b interactions inside TNAP active pocket

The nitrogen heteroatom of pyrrolo-naphthyridine ring of 1b formed a metallic linkage with Zn^2+^ metal, and the ring itself was involved in forming π-π linkage with His324 and π-cationic linkage with Arg167 and Asp320 residues. The tert-butyl phenyl ring formed π-σ linkage with His321 residue. Compound **1b** also showed van der Waals interactions with His154, Asp320, His321, His324, and His434 residues (Fig. 12).

**Fig. 13:**
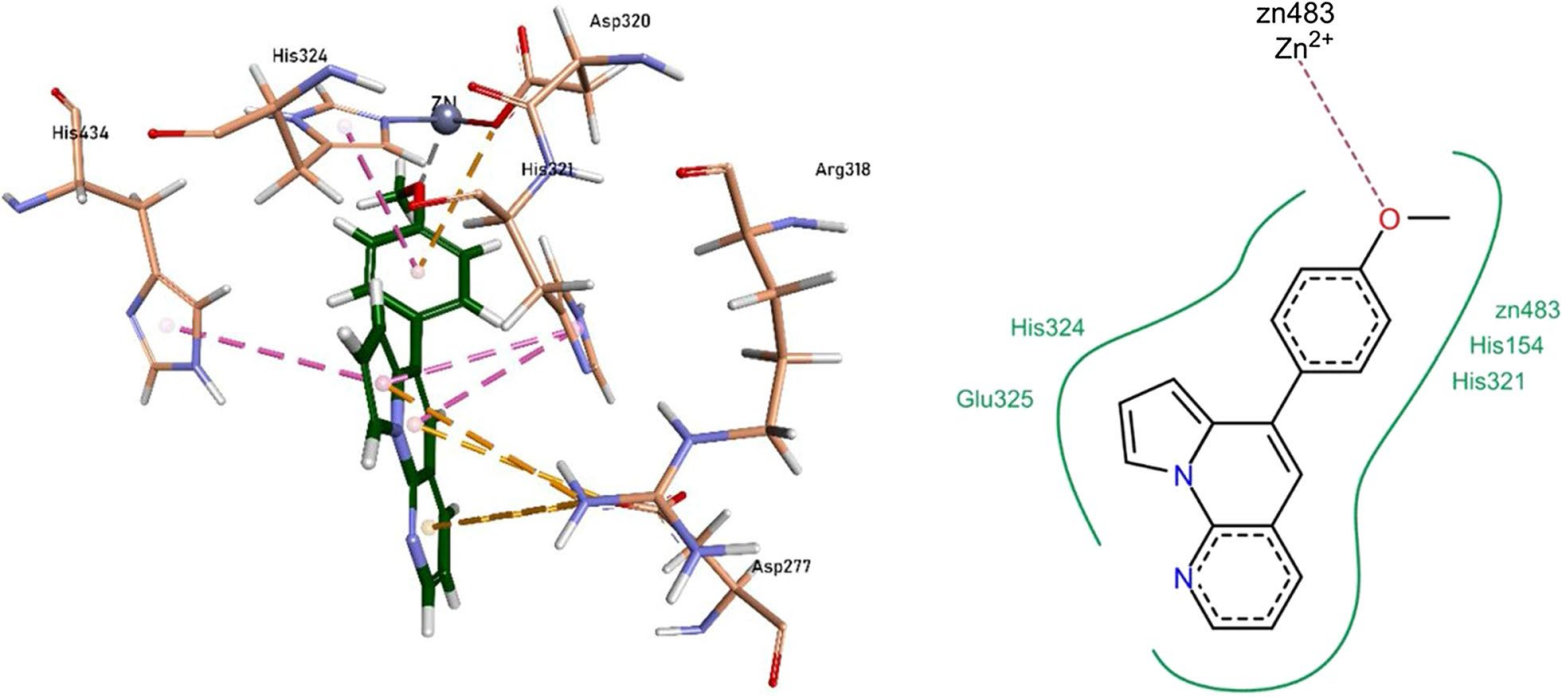
Compound 1f interactions inside TNAP active pocket

The pyrrolo-naphthyridine ring of 1f was involved in forming π-π linkages with His321 and His434 residues while π-cationic linkages with Asp277 and Arg318 residues. The methoxy moiety of methoxyphenyl ring formed a metallic linkage with Zn^2+^ metal, and the ring itself was involved in forming π-π linkage with His324 and π-cationic linkage with Asp320 residues. The compound **1f** also exhibited van der Waals interactions with His154, His321, His324, and Glu325 residues (Fig. 13).

### HYDE assessment of selective and potent test compounds against CA-II, CA-IX, CA-XII, *h*-IAP and *h*-TNAP

The HYDE affinity assessment tool of LeadIT was put into effect for the top 300 ranking docked conformations within the active sites of crystal structures of CA II, CA IX, CA XII, *h*-IAP and *h*-TANP.This affinityserved in the selection of appropriate binding mode of the most potent compound. The FlexX score of the selective compounds and their binding free energy ΔG are given in Table 5.

**Table 5:** HYDE assessment of the top-ranking poses

Code	FlexX score of the top-ranking pose	Binding free energy ΔG (kJ mol^−1^)
CA II		
1c	− 17.7028	− 26
1e	− 14.9324	− 16
1g	− 19.8175	− 27
CA IX		
1a	− 17.1683	− 18
1c	− 17.0636	− 14
1g	− 20.7607	− 25
1h	− 16.5644	− 35
CA XII		
1a	− 12.0087	− 29
1c	− 10.3354	− 28
1e	− 11.5124	− 30
1h	− 12.8028	− 24
1i	− 13.1051	− 29
1j	− 13.6699	− 29
*h*IAP		
1a	− 13.8338	− 7
1d	− 14.4856	− 4
1e	− 6.3932	− 5
1g	− 12.5653	− 18
1j	− 12.9547	− 6
1k	− 7.9856	− 19
*h*TNAP		
1a	− 9.8481	− 15
1b	− 11.3711	− 8
1e	− 8.5587	− 11
1f	− 14.4999	− 23
1k	− 11.0806	− 7
1l	− 10.2030	− 14

### Molecular dynamic simulations

After carrying out the docking studies, the binding results of the most potent derivatives were validated by MD simulation to augment the research findings further. The protein–ligand complexes of both proteins i.e. Carbonic Anhydrase (II and IX) and Alkaline Phosphatase with the most potent [1, 8]-Naphthyridine derivative for both groups of proteins i.e.**1g** was simulated for 100 ns in aqueous conditions by NAMD software. The Root Mean Square Deviation (RMSD) value and Root Mean Square Fluctuation (RMSF) value were used to analyze the conformational stability of proteins and their complexes. The RMSD graph details any structural variation caused by the ligand–protein interaction under a simulated environment. The results of MD simulation were assessed from the RMSD plots of both proteins as well as their derivatives. These RMSD plots were compared to evaluate the stability of the protein complexes. Further analysis of the MD simulation was done by plotting RMSF graphs, which is used to detect any changes in the target protein's C and N terminal lobe amino acid residues during the trajectory (see Additional file 1).

All the results of MD simulations were evaluated using RMSD graphs. The RMSD value less than 2 is considered good and indicates the stability of the ligand–protein complex.

### Carbonic anhydrase II with 1g

**Fig. 14:**
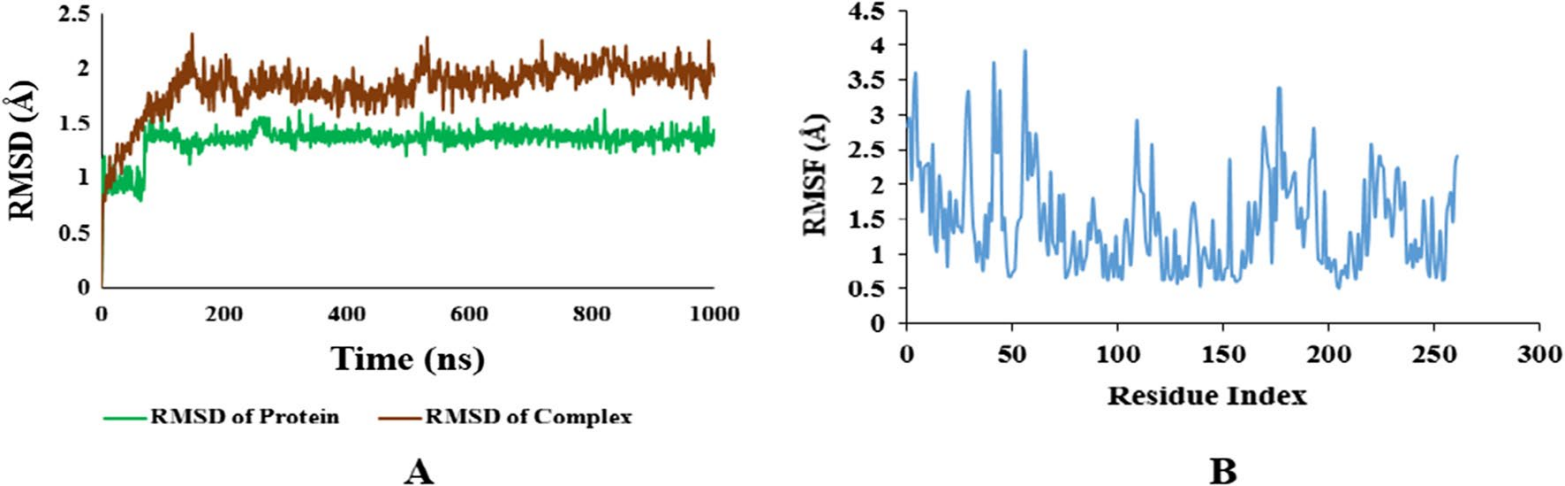
**A** RMSD plots of Protein and Protein–ligand complex of Carbonic Anhydrase II. **B** RMSF plots of protein–ligand complex

According to simulation results, the docking complex of carbonic anhydrase II with **1g** was found stable throughout the simulation time. The average RMSD value of protein is less than 2, which indicates the higher stability of the protein backbone, while the RMSD value of the protein–ligand complex was also smooth and within the acceptable range indicating its stability with an average RMSD value of 2 which is slightly higher than protein RMSD. In the same way, RMSF of the protein chain was also calculated, showing the slight fluctuation of Amino Acid Residues returning to its initial position with an average RMSF value of 1.5 Å. Results are elaborated in the following Fig. 14.

### Carbonic anhydrase IX with 1g

**Fig. 15:**
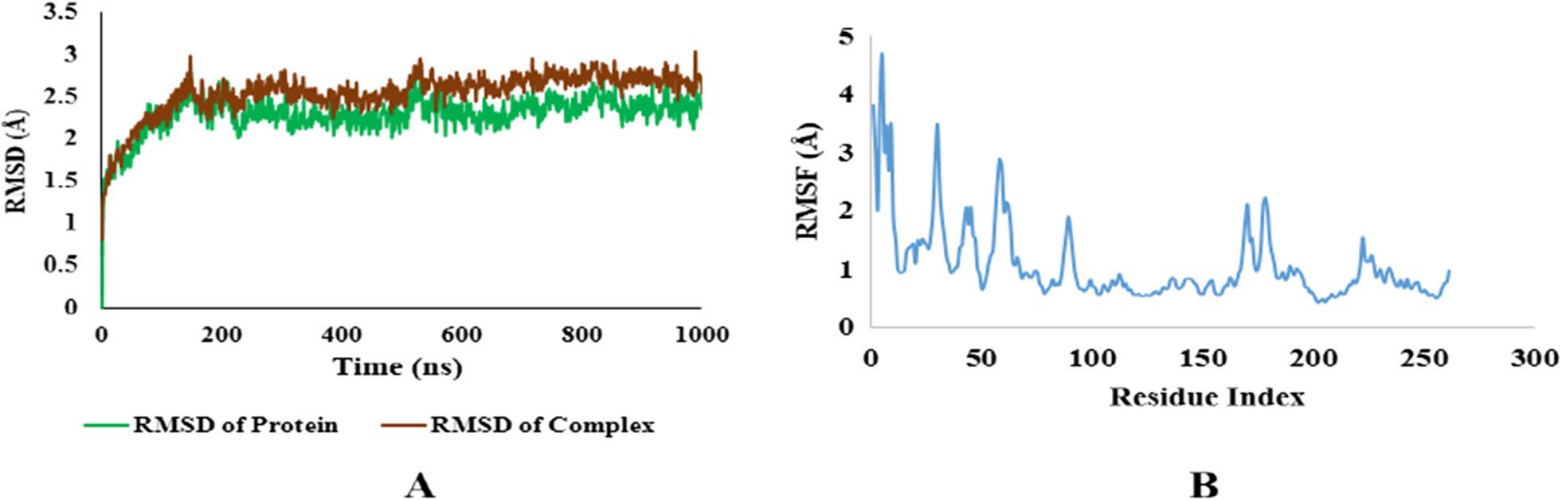
**A** RMSD plots of Protein and Protein ligand complex of Carbonic Anhydrase IX. **B** RMSF plots of protein ligand complex

In the same way docking complex of Carbonic anhydrase IX was also found stable throughout the simulation trajectory. The average RMSD value for the protein–ligand complex is slightly higher than 2 but is comparable to the protein RMSD graph indicating the stronger and stable association of the protein–ligand complex. The RMSF graph was also found stable with sight fluctuations which can be attributed to the flexibility of protein. The RMSD and RMSF graphs for carbonic anhydrase IX with **1g** are shown in the figure below. Results of molecular simulation are presented in Fig. 15.

### Alkaline phosphatase with 1g

**Fig. 16:**
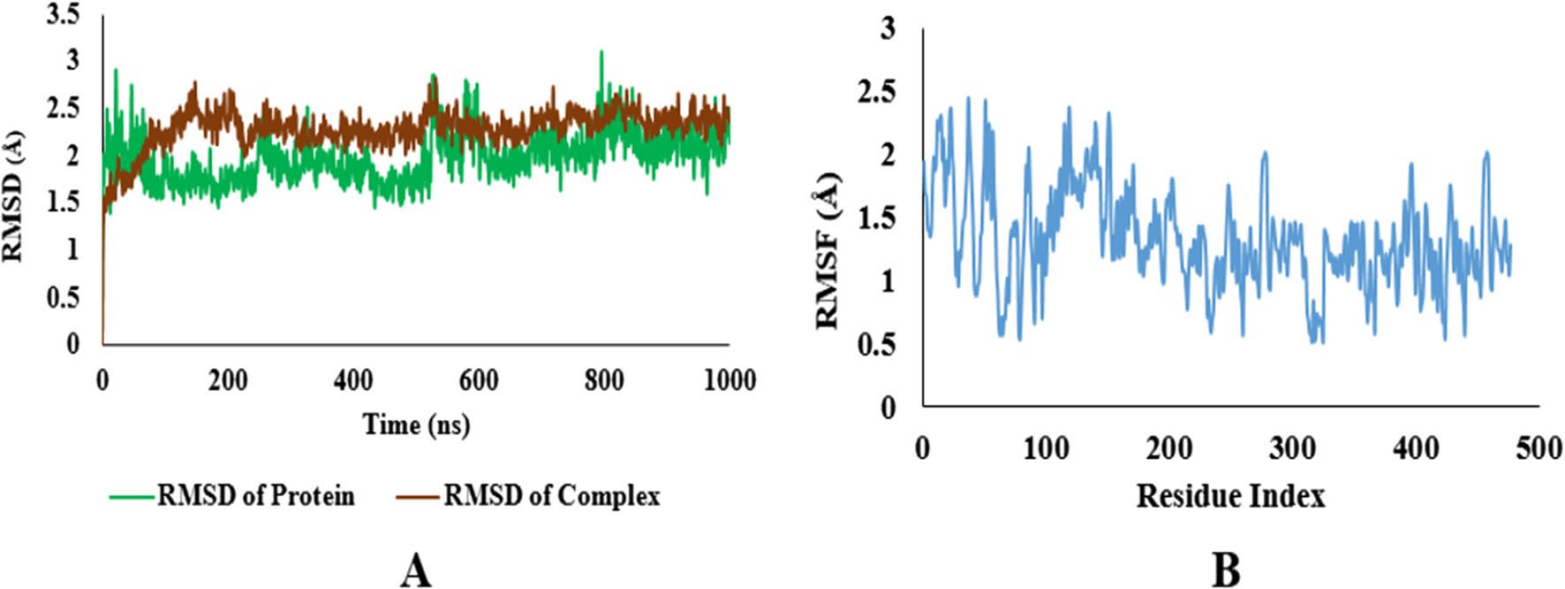
**A** RMSD plots of Protein and Protein ligand complex of alkaline phosphatase. **B** RMSF plots of protein ligand complex

The MD simulation results of protein alkaline phosphatase with **1g** also indicate stable interaction with an average RMSD value of 2.2 and are comparable to the protein RMSD value. The average RMSF value of 1.5 indicates the stability of protein residues during the trajectory (Fig. 16).

## Experimental

### Chemistry

**Fig. 17:**
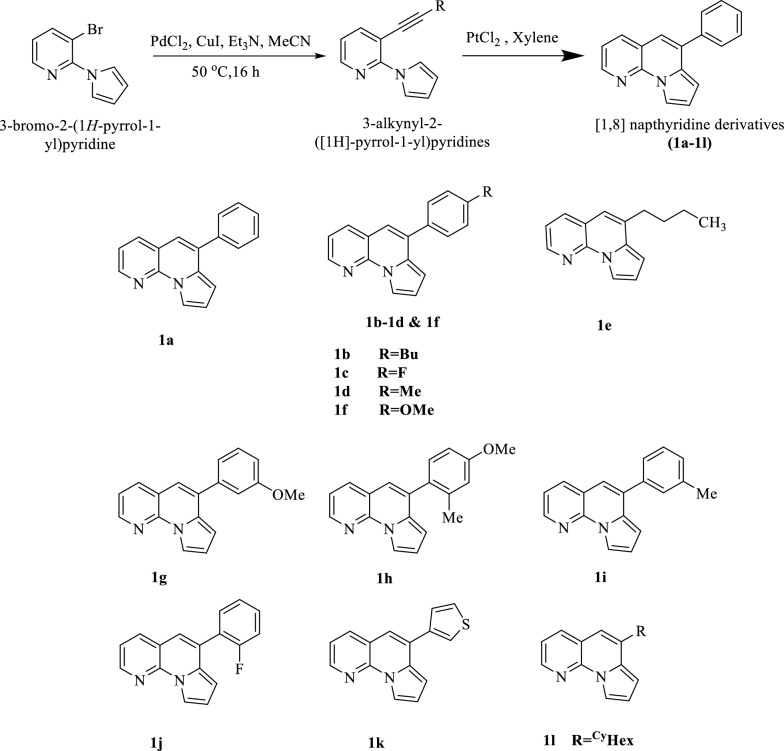
Synthesis scheme and derivatives of [1, 8]-Napthyridine derivatives (1a–1l) [26]

A general scheme and procedure for the synthesis of a novel series of [1, 8]-Napthyridine derivatives have been reported in a previous publication [28]. Sonogashira coupling reaction was used to synthesize the [1, 8]-Napthyridine derivatives. Initially, 3-bromo-2-(1H-pyrrol-1-yl)pyridine was dissolved in acetonitrile under an inert environment, then a catalyst PdCl_2_, CuI, and triethylamine were added to the reaction mixture. This reaction mixture was stirred at 50 °C for 24 h and synthesized 3-alkynyl-2-([1H]-pyrrol-1-yl)pyridine. 3-alkynyl-2-([1H]-pyrrol-1-yl)pyridine was cyclized in the presence of PtCl_2_(1.5 eq.) under argon in xylene at 120 °C for 24 h to give the desired [1, 8]-Naphthyridine derivatives. The reaction can be improved by using different Lewis acids and Brønsted acids. In addition, prolonged reaction time was applied, and PtCl_2_ was also used as a catalyst (Fig. 17).

### Expression of CA II, IX, and XII

Expression of enzyme protein was done according to previously reported protocol [29]. Briefly, transfection was carried out for CA-II, IX, and XII by using vectors pCMV3-N-His, pCMV3-SP-HIS-ORF, and pCMV3-N-His obtained from Sino Biologicals Inc., respectively. Vectors were stored at –20 °C, whereas, HEK 293 T was used for transfection_._ When cells gained about 80–90% confluency, they were washed twice with PBS and transfected with 0.2 µg plasmid solution. Cells were incubated for 6 h in 5% CO_2_ incubator, followed by the addition of fresh growth media. Cells were again incubated for 24 h. Hygromycin B (0.2 mg/mL) was used as a selection antibiotic for transfected cells. Cells were harvested after five days and lysed. Then desired proteins were purified via Ni–NTA resin using imidazole as affinity. SDS PAGE was carried out to confirm the molecular weight of purified protein.

### Inhibition assay for carbonic anhydrase

The CA isozymes inhibition assay was performed according to the reported method after some modifications [30]. Assay buffer was prepared that contained 50 mM tris-sulfate, 0.1 mM ZnCl_2,_ and pH was adjusted at 7.6. Initially, 60 µL assay buffer was added in each well of 96 wells plate, 10 µL test compound (final concentration 100 µM), and followed by the addition of 10 µL working enzyme solution. The reaction mixture was incubated for 10 min at 37 °C. Absorbance was measured as pre-read at 348 nm using a microplate reader (FLUOstar Omega, Germany). Then 20 µL of the substrate (6 mM) was added to the reaction mixture to initiate the reaction. The reaction mixture was incubated at 37 °C for 30 min, and absorbance was measured again. The activity was compared with a negative control, a reaction mixture without any inhibitor. Results were reported as the mean of triplicate experiments (± SEM). The % inhibition was calculated by following the formula.

$$ Inhibition~\left( \%  \right) = \left[ {100 - \left( {{\raise0.7ex\hbox{${OD~test~compound}$} \!\mathord{\left/ {\vphantom {{OD~test~compound} {OD~control}}}\right.\kern-\nulldelimiterspace} \!\lower0.7ex\hbox{${OD~control}$}}} \right) \times 100} \right] $$

### Alkaline phosphatase inhibition assay

[1, 8]-Naphthyridine derivatives were screened for inhibitor potential towards *b*-TNAP and *c*-IAP. The assay was based on previously reported protocol [31]. The assay volume was 100 µL, and the final concentration of each tested compound was 100 µM per well. All the solutions were made in assay buffer comprising Tris–HCl (50 mM), ZnCl_2_ (0.1 mM) and MgCl_2_ (5 mM) and pH was adjusted at 9.5. Working solutions of both enzymes were prepared in assay buffer containing 50% of glycerol. The final concentration of *b*-TNAP and *c*-IAP were kept at 0.025 U/mL and 0.05 U/mL, respectively. Assay was started by adding 70 µL of assay buffer in each well of 96 wells flat bottom clear microplate, followed by adding 10 µL of test compound and 10 µL of working enzyme solution. The reaction mixture was incubated at 37 °C for 10 min and absorbance was measured by a microplate reader (Bio-TekELx 800™, Instruments, Inc. USA) at 405 nm. The reaction was started by adding 10 µL of 5 mM *p*-NPP (para-nitrophenolphosphate) as substrate. After 30 min of incubation, absorbance was measured at percentage of enzyme inhibition was measured by comparing with a negative control that did not contain any inhibitor. The test compounds that exhibited a percentage of inhibition greater than 50% were further analyzed for dose–response curve, and IC_50_ values were calculated by PRISM 5.0 (GraphPad, San Diego, California, USA). Following formula was used to calculate the percentage of inhibition of test compounds.

$$ Inhibition~\left( \%  \right) = \left[ {100 - \left( {{\raise0.7ex\hbox{${OD~test~compound}$} \!\mathord{\left/ {\vphantom {{OD~test~compound} {OD~control}}}\right.\kern-\nulldelimiterspace} \!\lower0.7ex\hbox{${OD~control}$}}} \right) \times 100} \right] $$

### Density functional theory (DFTs)

For all the DFT calculations, Guassian09 programme [32] was used with the B3LYP functional scheme and 3-21G basis set to carry out the density functional theory (DFTs) calculations [33]. The Computational calculation of electronic structure for atoms and molecules makes use of this efficient theory. The chemical structures of all the chosen derivatives were drawn in Chemdraw 12.0 to facilitate DFT experiments. ChemDraw 3D Pro was used in a similar manner for superficial energy minimization, and files were saved in the common Sybyl mol2 format. Frontier molecular orbital (FMO), global, and local reactivity descriptors were obtained, together with the optimized geometric parameters. Fchk files were analyzed with Guass View 6 [34].

## Molecular docking studies

### Selection of the protein structures and preparation of ligands

Crystallographic structures of CA-II, CA-IX, and CA-XII with PDB IDs 3K34, 6G9U, and 5MSA, respectively, were procured from the Protein Data Bank. The x-ray crystal structures of *h*-TNAP and *h*-IAP are currently not present in the Protein Data Bank; homology-modeled already reported structures from our group have resorted to docking.

The crystal structures were prepared utilizing the Protein Preparation Wizard, which was implemented within the MOE software, employing the default settings. Hydrogen atoms were incorporated into the molecular operating environment (MOE) utilizing the protonate 3D protocol. Subsequently, the protein structures underwent minimization employing the MMFF94x force field until reaching an RMSD gradient of 0.1 kcal·mol^−1^ Å^−1^ [35]. Structures of selected compounds and their stereochemistry was patched by energy minimization. *h*-TNAP and *h*-IAP models did not have any incorporated ligands, so the validation of these enzymes models was done with the positive standards used in the biological assay. Inbuilt MOE site finder was applied for the selection of possible binding sites of the target protein. Zn^2+^ was kept in the center of enzyme-selected active sites. After initial validation, molecular docking of the targeted compounds was performed.

### Docking of ligands in targeted proteins

The docking studies were executed by docking software LeadIT [36]. Software default parameters were used to perform the molecular docking studies of the reference standards that were used in in vitro assay as well as for selected compounds. For each ligand, the most promising docked pose was selected and was further assessed through the HYDE assessment tool. The Discovery Studio Visualizer (v19.1.0.18287) was used to represent 3D interaction of selected docked poses [37].

### Molecular dynamics simulation studies

The molecular dynamic simulation studies were performed using Nanoscale molecular dynamic (NAMD) software on a CUDA-accelerated GPU machine with a 16-core CPU and 64 GB RAM memory with top-ranked conformation. The visualization of results was done using VMD software [38]. The MD simulation is used to ascertain the binding interactions with targeted proteins as well as stability of the protein–ligand complex under accelerated conditions. The top-ranked protein–ligand complex was selected, and topology files for the protein and the ligand were created using the CHARMM36 forcefield [39]. The required NaCl charges were added to neutralize the system. To eliminate any close atomic interactions, the system was reduced to the sharpest energy gradient. The system was equilibrated in an NVT ensemble for 500,000 steps, followed by another 500,000 steps in an NPT ensemble. After that, a simulation with specific periodic boundaries was run for 100 ns [40].

## Conclusion

In the summary, [1,8]-Naphthyridine derivatives **(1a-1l)** were found to be potent inhibitors of carbonic anhydrase as well as alkaline phosphatase isozymes. Among the test compounds, the most potent inhibitors for CA-II, CA-IX, and CA-XII were **1e**, **1g**, and **1a** with IC_50_values of 0.44 ± 0.19, 0.11 ± 0.03 and 0.32 ± 0.07 µM, respectively. While in the case of ALPs, the most potent compounds for *b*-TNAP and *c*-IAP were **1b** and **1e** with IC_50_ values of 0.122 ± 0.06 and 0.107 ± 0.02 µM, respectively. In silico studies, including Density Function Theory, Molecular docking, and MD simulations, confirmed strong binding interactions exist between active sites of enzymes and potential inhibitors. These dual inhibitors of both the enzymes may prove to be very efficient in the treatment of various bone disorders, especially rheumatoid arthritis. Based on this study, more potent molecules can be designed as dual inhibitors of both enzymes for further investigation in the future.

### Supplementary Information

**Additional file 1: Figure S1.** Compound 1c interactions inside CA-II (3k34) active pocket. **Figure S2.** Compound 1e interactions inside CA II (3k34) active pocket. **Figure S3.** Compound **1a** interactions inside CA-IX (6G9U) active pocket. **Figure S4.** Compound **1c** interactions inside CA-IX (6G9U) active pocket. **Figure S5.** Compound **1h** interactions inside CA-IX (6G9U) active pocket. **Figure S6.** Compound **1c** interactions inside CA-XII (5MSA) active pocket. **Figure S7.** Compound **1e** interactions inside CA-XII (5MSA) active pocket. **Figure S8.** Compound **1h** interactions inside CA-XII (5MSA) active pocket. **Figure S9.** Compound **1e** interactions inside hIAP active pocket. **Figure S10.** Compound **1g** interactions inside hIAP active pocket. **Figure S11.** Compound **1j** interactions inside hIAP active pocket. **Figure S12.** Compound **1k** interactions inside hIAP active pocket. **Figure S13.** Compound **1a** interactions inside TNAP active pocket. **Figure S14.** Compound **1e** interactions inside TNAP active pocket. **Figure S15.** Compound **1k** interactions inside TNAP active pocket. **Figure S16.** Compound **1l** interactions inside TNAP active pocket.

## Data Availability

Data will be available on request by the corresponding author.
