# Supplementary material for: [1, 8]-Naphthyridine derivatives as dual inhibitor of alkaline phosphatase and carbonic anhydrase
Source: BMC Chem. 2023 Oct 25;17(1):142. doi: 10.1186/s13065-023-01052-8 (PMC10599030; doi:10.1186/s13065-023-01052-8)
Supplement: Supplementary file 1 — Additional file 1: Figure S1. Compound 1c interactions inside CA-II (3k34) active pocket. Figure S2. Compound 1e interactions inside CA II (3k34) active pocket. Figure S3. Compound 1a interactions inside CA-IX (6G9U) active pocket. Figure S4. Compound 1c interactions inside CA-IX (6G9U) active pocket. Figure S5. Compound 1h interactions inside CA-IX (6G9U) active pocket. Figure S6. Compound 1c interactions inside CA-XII (5MSA) active pocket. Figure S7. Compound 1e interactions inside CA-XII (5MSA) active pocket. Figure S8. Compound 1h interactions inside CA-XII (5MSA) active pocket. Figure S9. Compound 1e interactions inside hIAP active pocket. Figure S10. Compound 1g interactions inside hIAP active pocket. Figure S11. Compound 1j interactions inside hIAP active pocket. Figure S12. Compound 1k interactions inside hIAP active pocket. Figure S13. Compound 1a interactions inside TNAP active pocket. Figure S14. Compound 1e interactions inside TNAP active pocket. Figure S15. Compound 1k interactions inside TNAP active pocket. Figure S16. Compound 1l interactions inside TNAP active pocket. [file 13065_2023_1052_MOESM1_ESM.docx]

**[1,8]-Naphthyridine derivatives as dual inhibitor of alkaline phosphatase and carbonic anhydrase**

Salman Alrokayan^1,2^ Tajamul Hussain^1,3^, Salman Alamery^2^, Arif Ahmed Mohammed^2^, Abid Mahmood^4^, Syeda Abida Ejaz^5^, Peter Langer^6^, Jamshed Iqbal^4,*^

*^1^Research Chair for Biomedical Application of Nanomaterials. Biochemistry Department, College of Science, King Saud University, Riyadh, Saudi Arabia 11451.*

*^2^Biochemistry Department, College of Science, King Saud University, Riyadh, Saudi Arabia 11451*

*^3^Centre of Excellence in Biotechnology Research, King Saud University, Riyadh, Saudi Arabia 11451*

*^4^Centre for Advanced Drug Research, COMSATS University Islamabad, Abbottabad Campus, Abbottabad-22060, Pakistan*

*^5^Department of Pharmaceutical Chemistry, Faculty of Pharmacv, The Islamia University of Bahawalpur, Bahawalpur, 63100, Pakistan*

*^6^Universität Rostock, Institut für Chemie, A.-Einstein-Str. 3a, 18059 Rostock, Germany*

**Corresponding Author**

Prof. Dr. Jamshed Iqbal ([drjamshed@cuiatd.edu.pk](mailto:drjamshed@cuiatd.edu.pk); [jamshediqb@gmail.com](mailto:jamshediqb@gmail.com))

**Molecular docking studies:**

**Molecular docking studies against carbonic anhydrase II (3K34)**

The *in vitro* analysis revealed that compounds **1c**, **1e**, and **1g** were potent inhibitors of carbonic anhydrase II. The nitrogen heteroatom of pyrrolo-naphthyridine ring of 1c formed a metallic linkage with Zn^2+^ metal, and the same pyrrolo-naphthyridine ring was involved in hydrogen bonding with His94, His119, and Thr199, while forming π-π linkages with Val143 and Leu198. The compound **1c** also exhibited van der Waals interactions with His96, Phe131, Leu198, and Thr200 (Figure. 01).


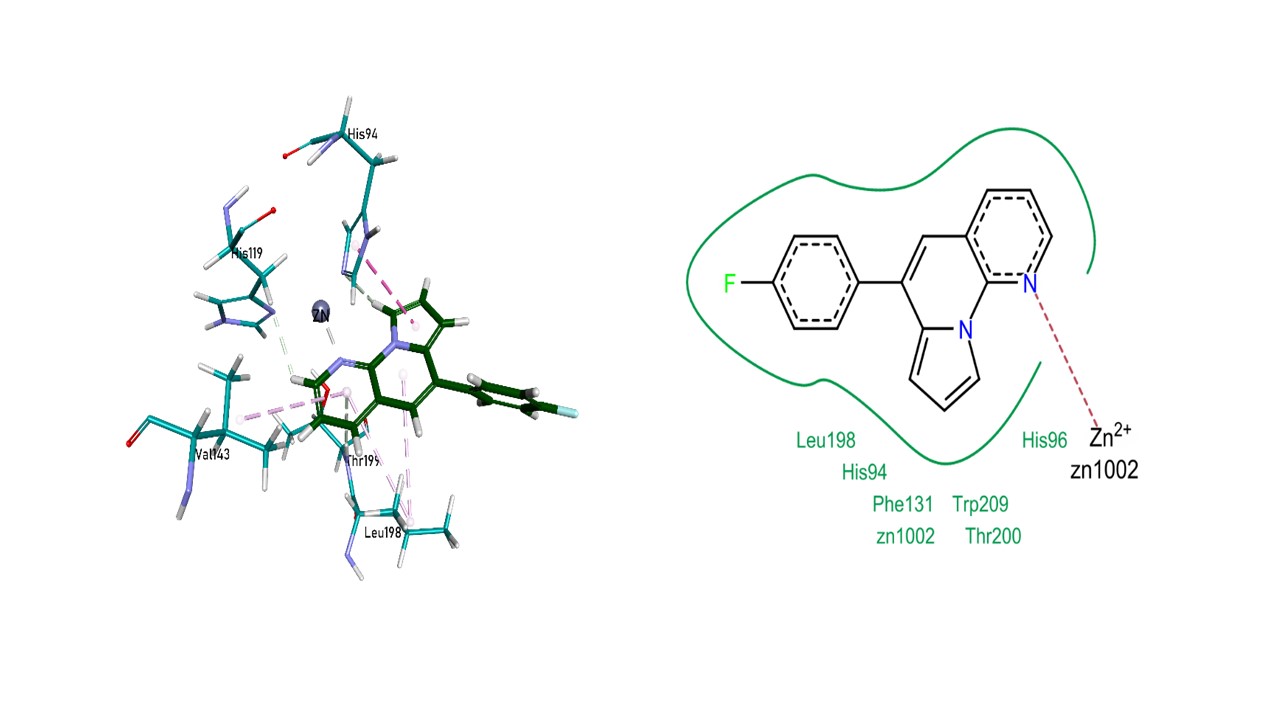


**Supplementary Figure 01** Compound **1c** interactions inside CA-II (3k34) active pocket

The pyrrolo-naphthyridine ring of 1e formed a π-cationic bond with Zn^2+^ metal, and the same pyrrolo-naphthyridine ring was involved in hydrogen bonding with Thr199 and Thr200, while forming π-π linkages with His94, Val121, Val143, and Leu198. Butyl moiety on the compound 1e was involved in π-alkyl linkage with Phe131 residue. The compound 1e also exhibited van der Waals interactions with His94, His119, Val121, Phe131, Val143, and Leu198 (Figure 02).


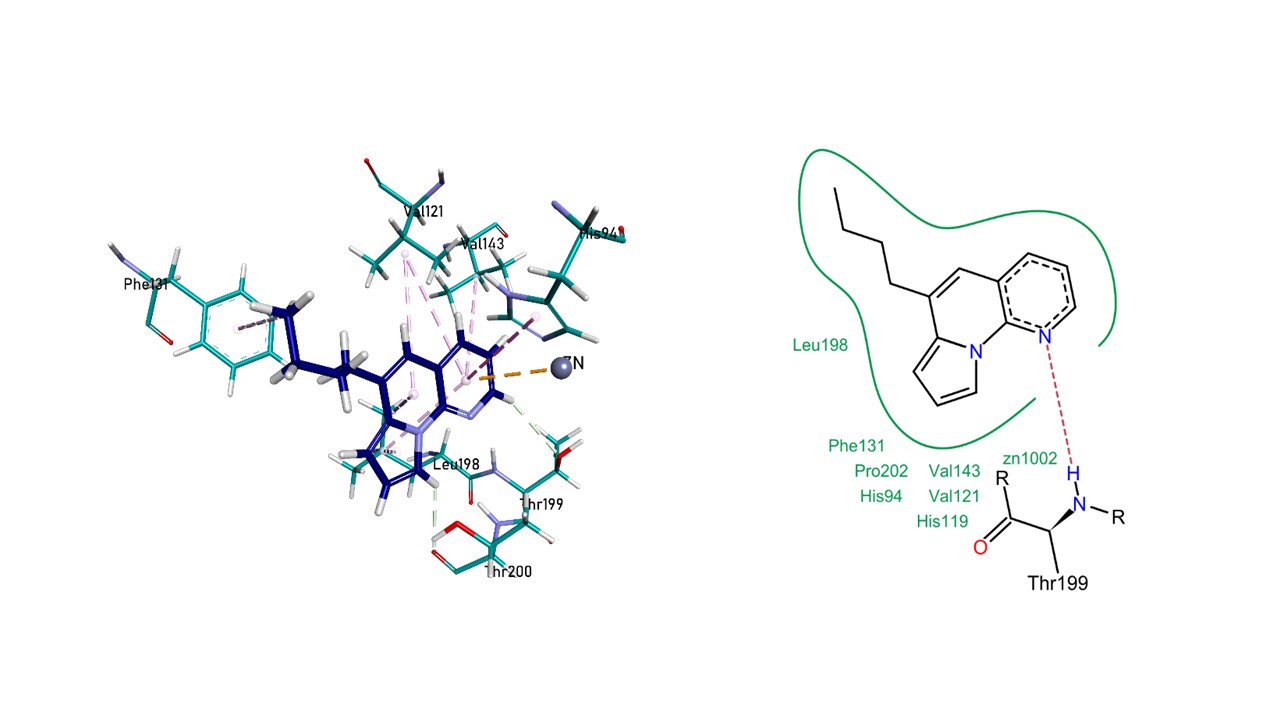


**Supplementary Figure 02** Compound 1e interactions inside CA II (3k34) active pocket

**Molecular docking studies against carbonic anhydrase IX (6G9U)**

The biological assay revealed **1a, 1c, 1g**, and **1h** to be the most potent inhibitors for the carbonic anhydrase IX enzyme. The nitrogen heteroatom of pyrrolo-naphthyridine ring of **1a** formed a

metallic linkage with Zn^2+^ metal and a hydrogen bonding with Thr199, while forming π-π linkages with Val121 and Leu198. Phenyl moiety on the compound 1a was involved in π-alkyl linkages with Leu91, Val121 and Val131 residues. The compound 1a also exhibited van der Waals interactions with Leu91, Gln92, His94, Val121, Val131, Val143, Leu198 and Thr200 (Figure 03).


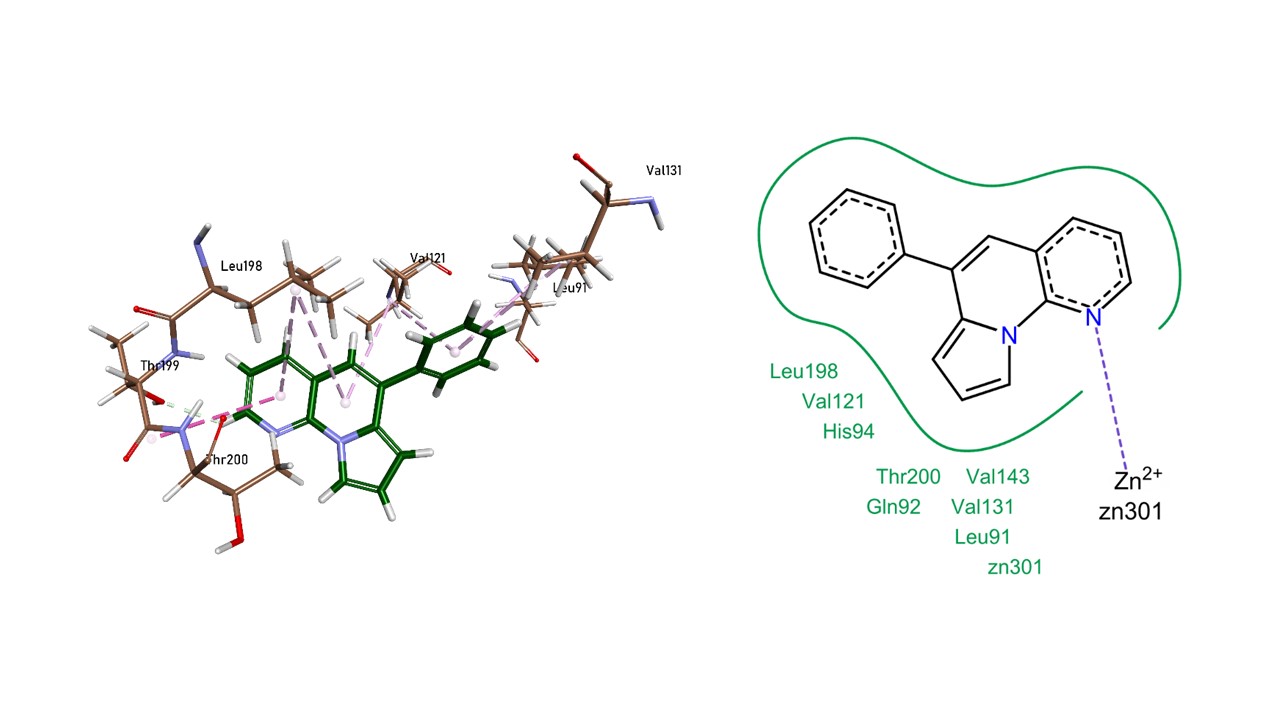


**Supplementary Figure 03** Compound **1a** interactions inside CA-IX (6G9U) active pocket

The nitrogen heteroatom of pyrrolo-naphthyridine ring of 1c formed a metallic linkage with Zn^2+^ metal and a hydrogen bonding with Gln92, while forming π-π linkages with Val121 and Leu198. Fluorophenyl moiety of the compound 1c was involved in π-alkyl linkages with Val121 and Val131 residues. The compound 1c also exhibited van der Waals interactions with Leu91, Gln92, His94, Val121, Val131, Leu198 and Thr200 (Figure 04).


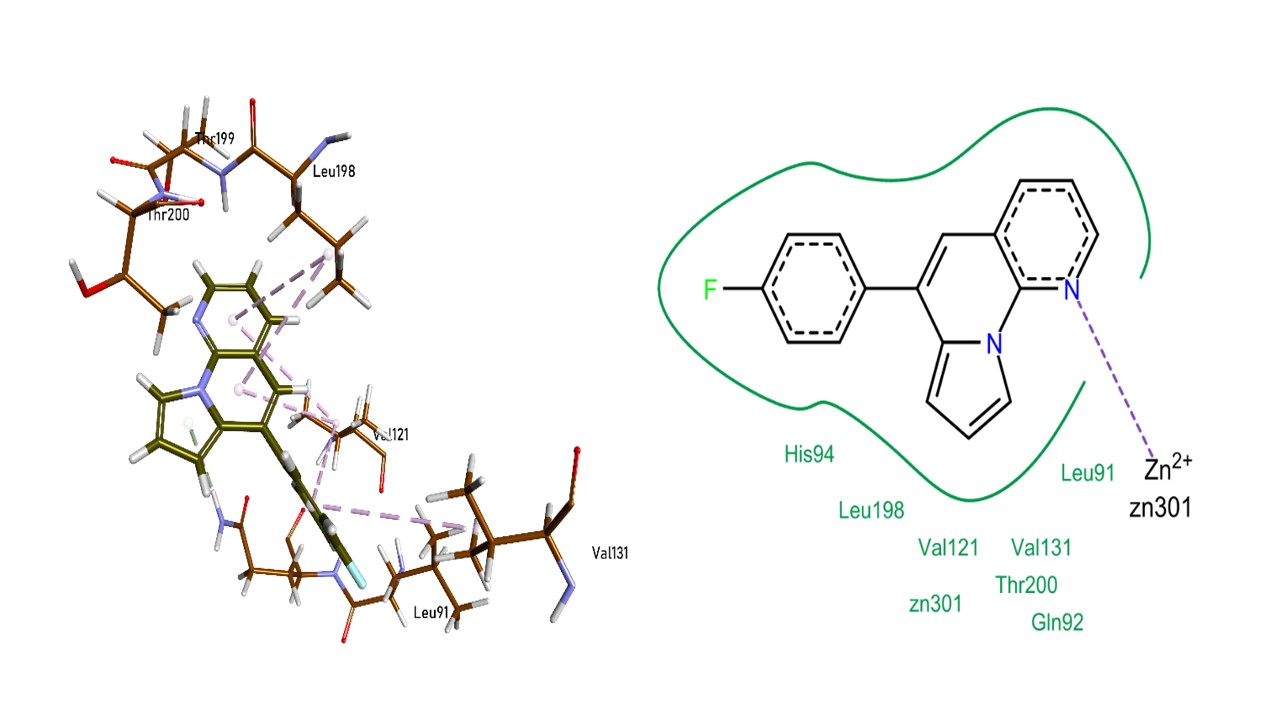


**Supplementary Figure 04** Compound **1c** interactions inside CA-IX (6G9U) active pocket

The methoxy moiety on the methoxyphenyl ring of 1h formed a metallic bond with Zn^2+^ metal, and the pyrrolo-naphthyridine ring of 1h formed π-π linkages with Val121, Val131, and Leu198. The methoxyphenyl ring of the compound 1g was involved in π-alkyl linkages with His94 and Leu198 residues. The compound 1g also exhibited van der Waals interactions with Leu91, Gln92, His94, Val121, Val131, Leu198 and Thr200 (Figure 05).


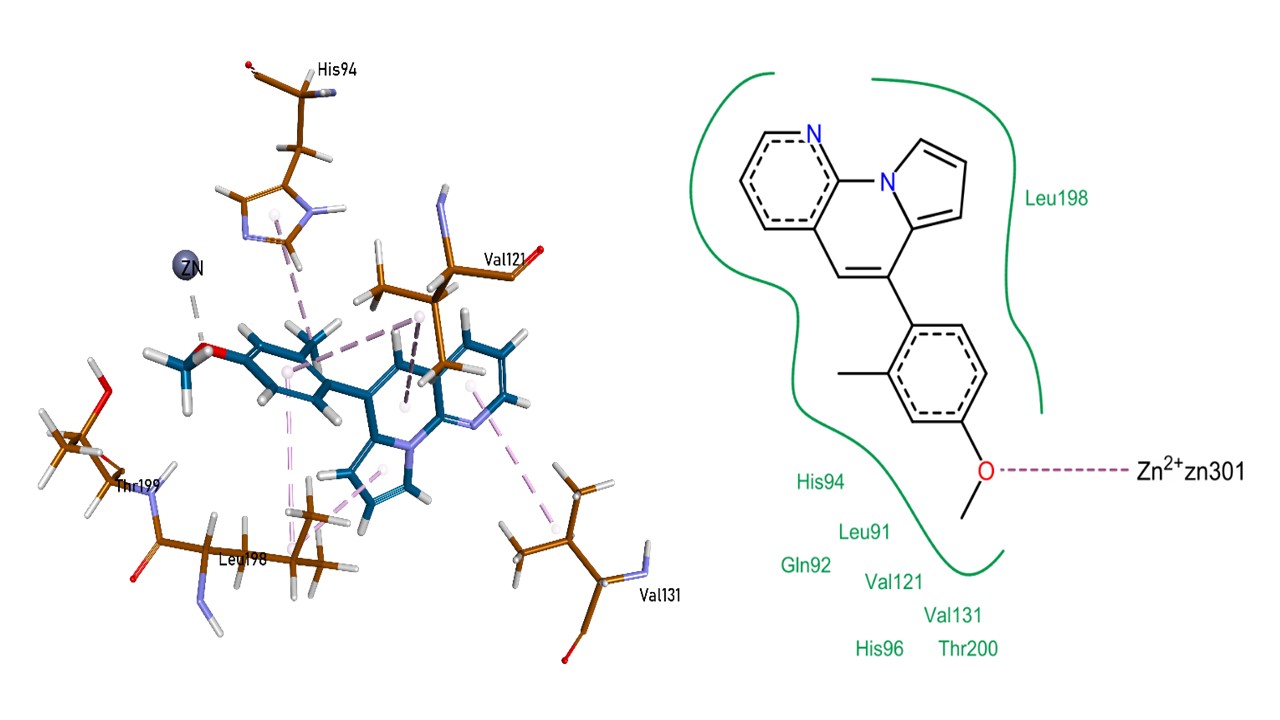


**Supplementary Figure 05** Compound **1h** interactions inside CA-IX (6G9U) active pocket

**Molecular docking studies against carbonic anhydrase-XII (5MSA)**

The nitrogen heteroatom of pyrrolo-naphthyridine ring of 1c formed a metallic linkage with Zn^2+^ metal and forming π-π linkages with His91, while π-alkyl bonding with Leu197 residue. Pyrrolo-naphthyridine ring was also involved in the formation of hydrogen bonding with Thr198 residue. The compound **1c** also exhibited van der Waals interactions with His91, His93, Leu197, and Thr199 residues (Figure 06).


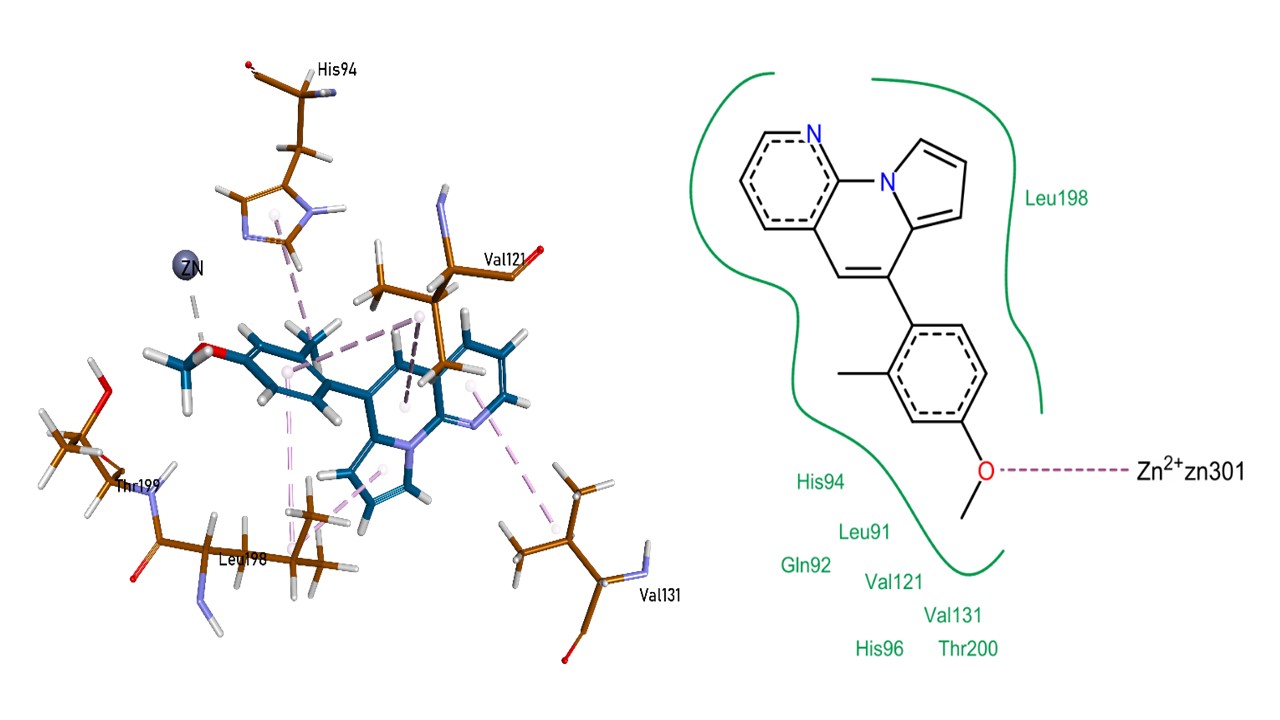


**Supplementary Figure 06** Compound **1c** interactions inside CA-XII (5MSA) active pocket

The nitrogen heteroatom of pyrrolo-naphthyridine ring of 1e formed a metallic linkage with Zn^2+^ metal and forming π-π linkages with His91, while π-alkyl bonding with Leu197 residue. Pyrrolo-naphthyridine ring was also involved in forming hydrogen bonding with Thr198 residue. The butyl moiety interacted with Pro201 residue through π-alkyl linkage. The compound **1e** also exhibited van der Waals interactions with His91, His93, Val119, Leu197, and Thr199 residues (Figure 07).


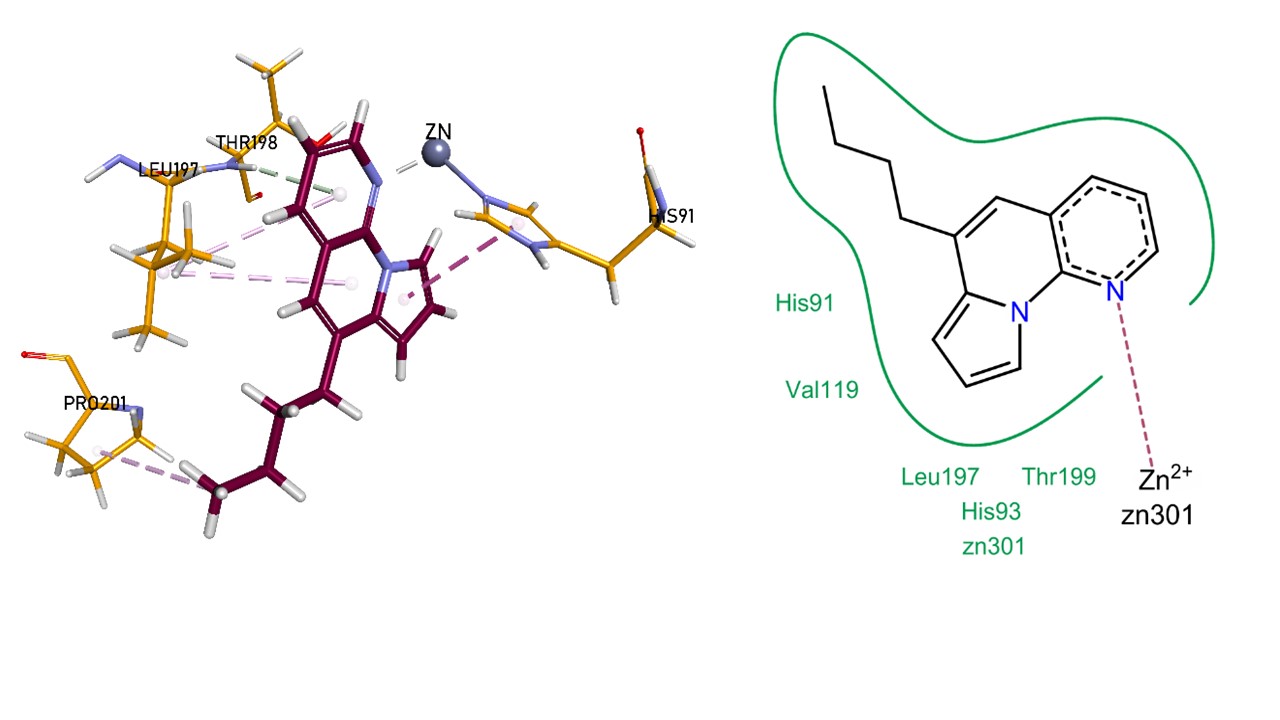


**Supplementary Figure 07** Compound **1e** interactions inside CA-XII (5MSA) active pocket

The nitrogen heteroatom of pyrrolo-naphthyridine ring of 1h formed a metallic linkage with Zn^2+^ metal and forming π-π linkages with His91, while π-alkyl bonding with Leu197 residue. Pyrrolo-naphthyridine ring was also involved in forming hydrogen bonding with Thr198 residue. The compound 1h also exhibited van der Waals interactions with His91, Leu197, Thr199, Pro201 and Trp208 residues (Figure 08).


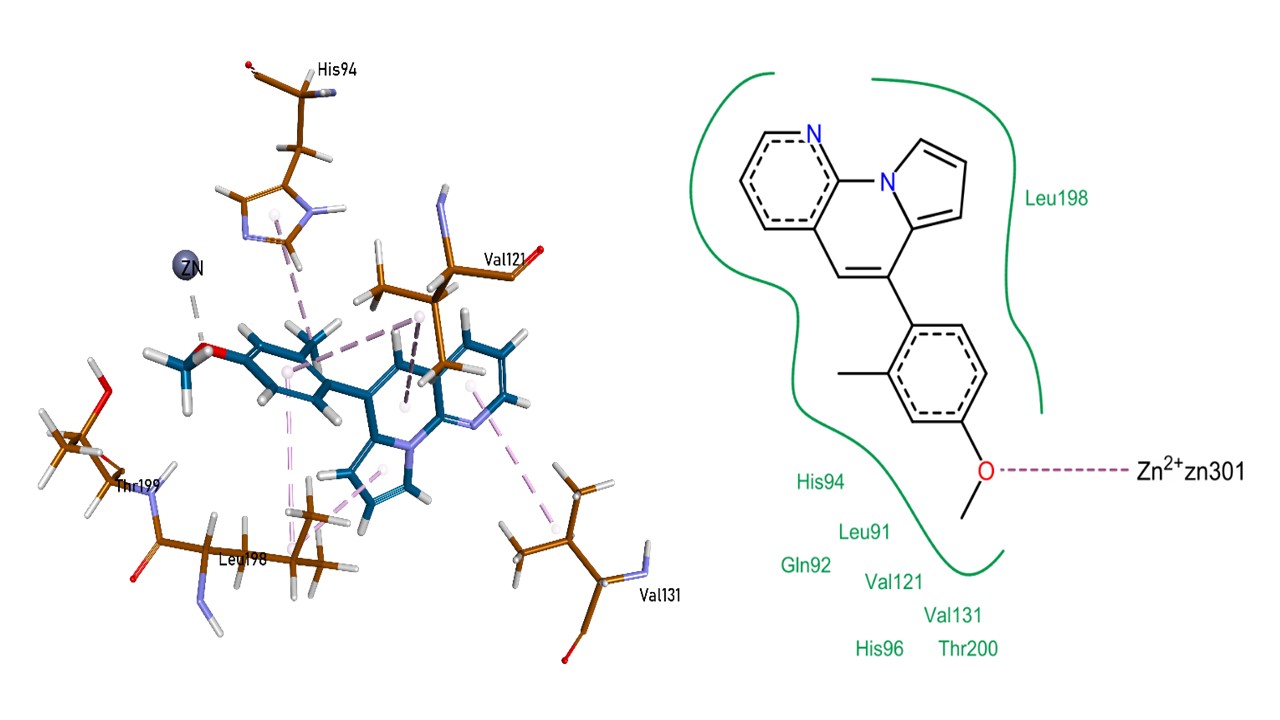


**Supplementary Figure 08** Compound **1h** interactions inside CA-XII (5MSA) active pocket

**Molecular docking studies against human intestinal alkaline phosphatase (*h*-IAP)**

The nitrogen heteroatom of pyrrolo-naphthyridine ring of 1e formed hydrogen bonding with His317 and His153 residues, and the pyrrolo-naphthyridine ring itself was involved in the formation π-cationic bonding with Arg166 and Zn^2+^ metal of the active pocket. The compound **1e** also exhibited van der Waals interactions with Asp316, His317, and His320 protein residues (Figure 09).


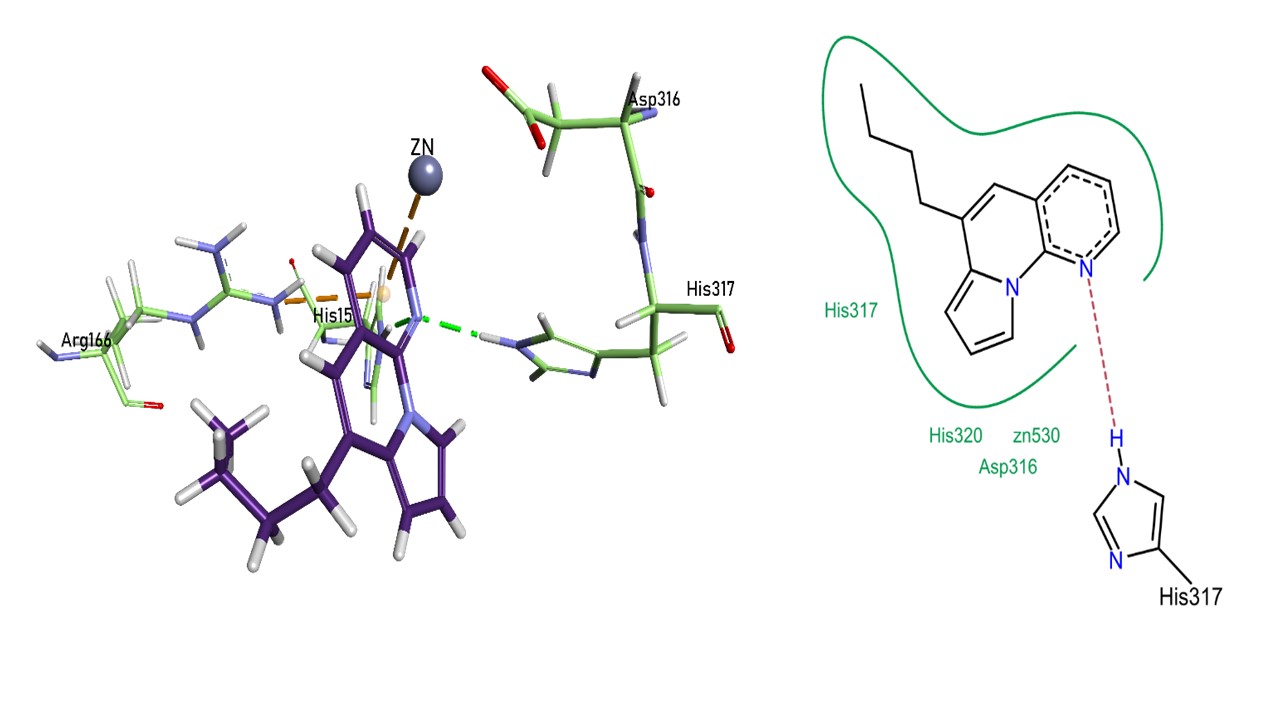


**Supplementary Figure 09** Compound **1e** interactions inside hIAP active pocket

The methoxy moiety on the methoxyphenyl ring of 1g formed a metallic bond with Zn^2+^ metal and the ring itself formed π-π linkages with His320, and π-cationic interaction with Arg166 residue. The compound **1g** also exhibited van der Waals interactions with Phe107, His317, His320, and His432 protein residues (Figure 10).


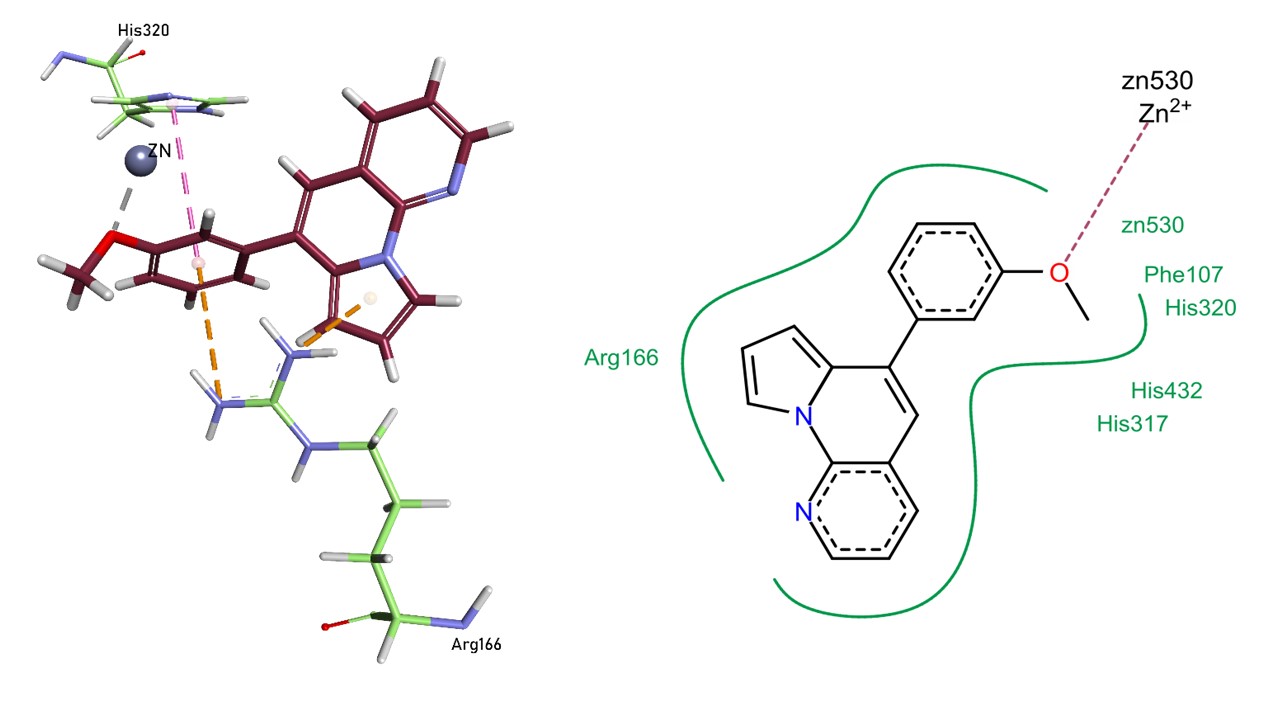


**Supplementary Figure 10** Compound **1g** interactions inside hIAP active pocket

The nitrogen heteroatom of pyrrolo-naphthyridine ring of 1j formed hydrogen bonding with His317 residue, and the pyrrolo-naphthyridine ring itself was involved in the formation π-cationic bonding with Arg166 and Zn^2+^ metal of the active pocket. The compound 1j also exhibited van der Waals interactions with Asp42, Ser92, Arg166, Asp316, His317, His320, and His432 residues of the protein (Figure 11).


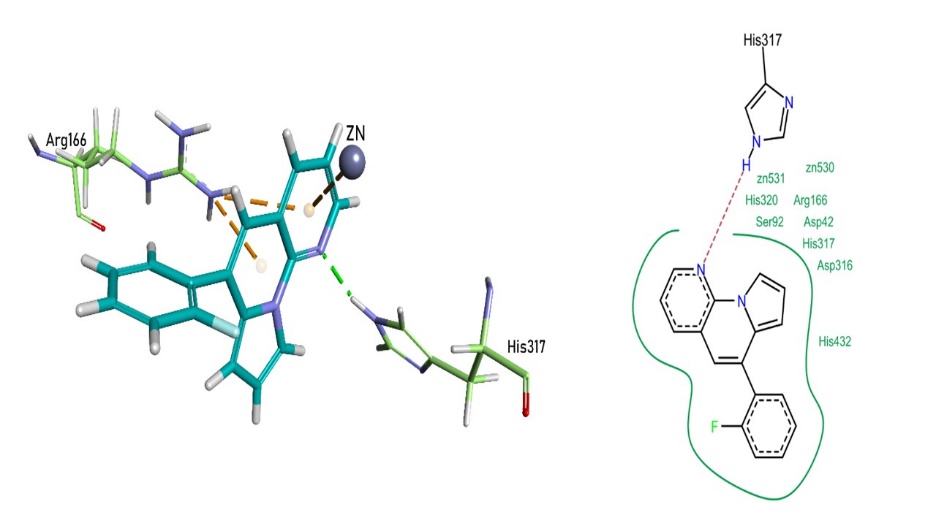


**Supplementary Figure 11** Compound **1j** interactions inside hIAP active pocket

The sulphur heteroatom from thiophene residue of 1k formed a metallic bond with Zn^2+^ metal and the pyrrolo-naphthyridine ring formed π-π linkages with His317 residue. The compound 1k also exhibited van der Waals interactions with His317 and His320 residues of the protein (Figure 12).


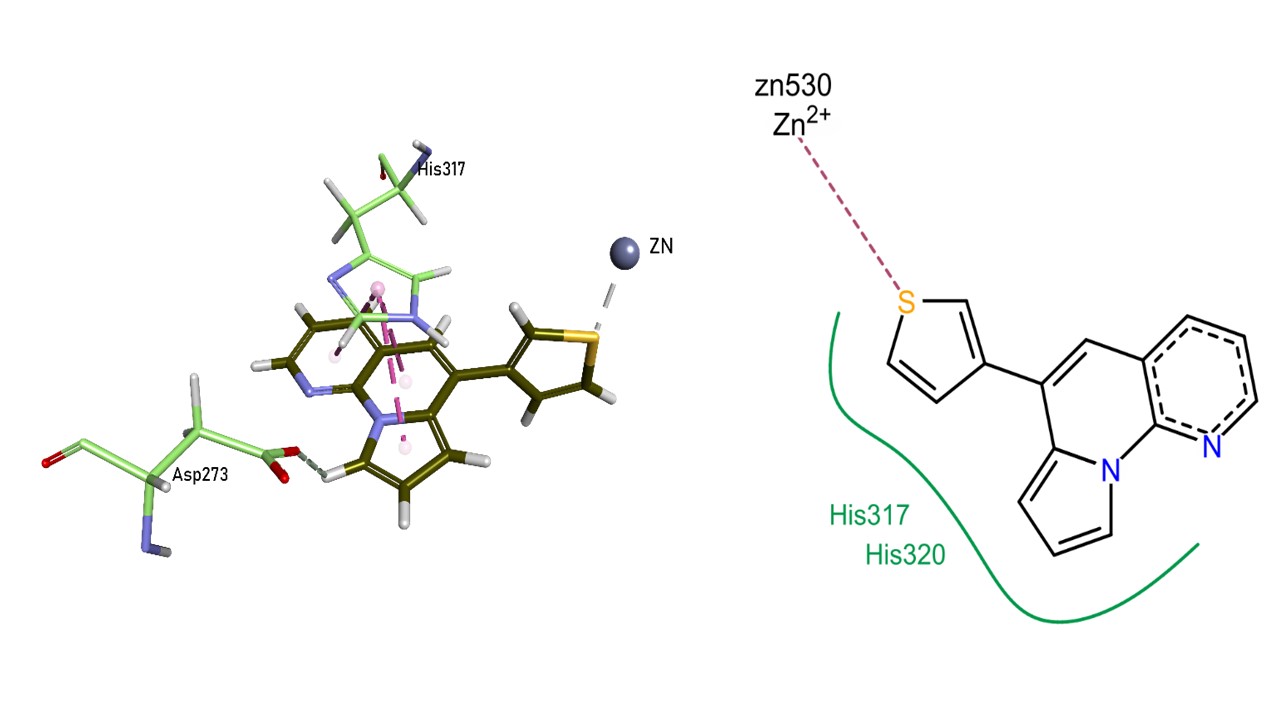


**Supplementary Figure 12** Compound **1k** interactions inside hIAP active pocket

**Molecular docking studies against human tissue non-specific alkaline phosphatase (*h*-TNAP)**

The nitrogen heteroatom of pyrrolo-naphthyridine ring of **1a** formed a metallic linkage with Zn^2+^ metal and the ring itself was involved in forming π-π linkage with His324 and π-cationic linkage with Arg167 residue. The compound **1a** also exhibited van der Waals interactions with His154, Arg167, His321, His324, and His437 residues (Figure 13).


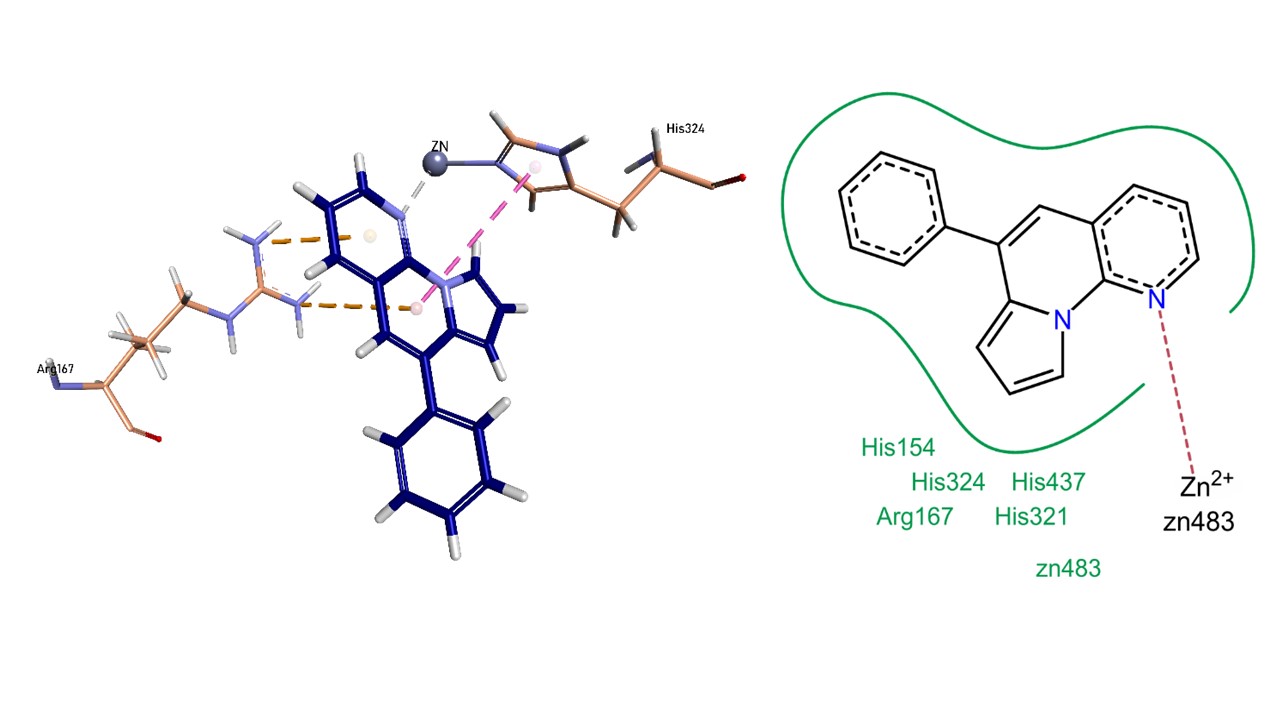


**Supplementary Figure 13** Compound **1a** interactions inside TNAP active pocket

The nitrogen heteroatom of pyrrolo-naphthyridine ring of 1e formed a metallic linkage with Zn^2+^ metal, and the ring itself was involved in forming π-π linkage with His324 and π-cationic linkage with Arg167 and Asp320 residues. The butyl moiety formed π-alkyl linkage with His434 residue. The compound **1e** also exhibited van der Waals interactions with His154, Asp320, His321, His324, and His434 residues (Figure 14).


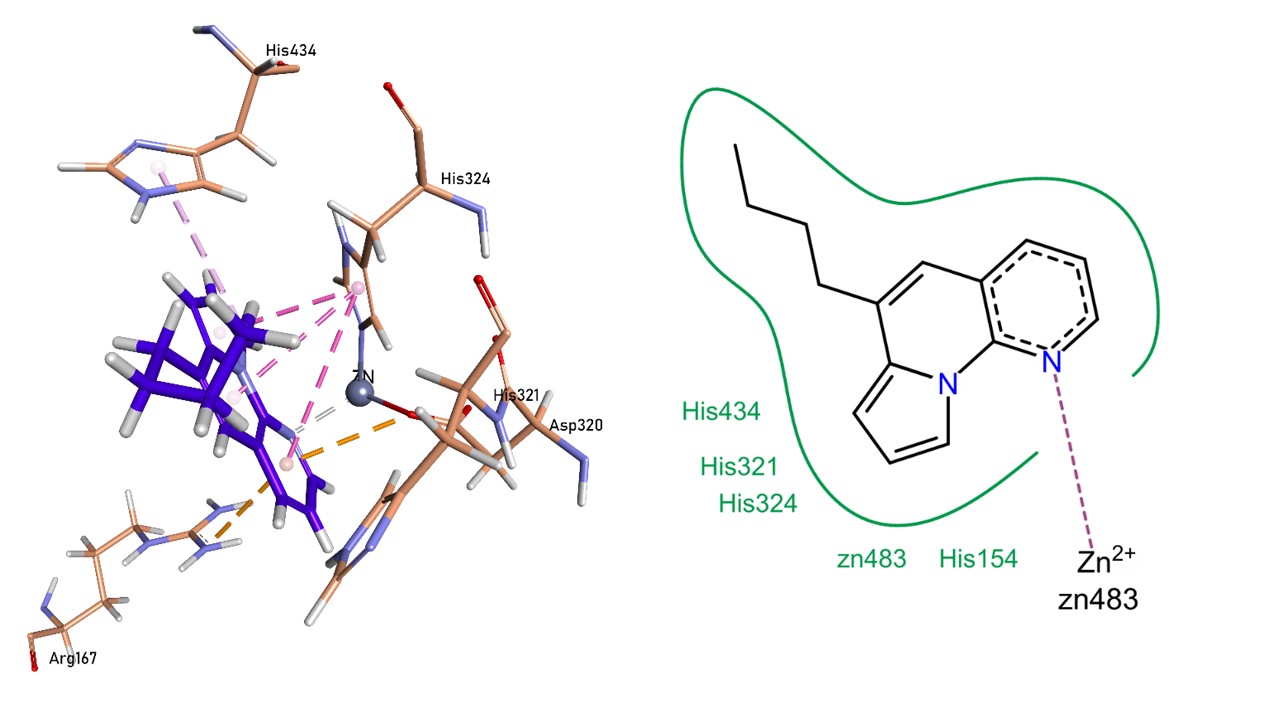


**Supplementary Figure 14** Compound **1e** interactions inside TNAP active pocket

The sulphur heteroatom from thiophene residue of 1k formed a metallic bond with Zn^2+^ metal and showed π-sulfur interaction with His154 and His437 residues, while the ring itself formed π-π linkage with His324 residue. The nitrogen heteroatom of pyrrolo-naphthyridine ring of 1k formed a hydrogen bond with Arg151 and the ring was involved in forming π-π linkage with His154 and π-anionic linkage with Asp277 residues. The compound 1k also exhibited van der Waals interactions with Arg167, Asp277, and His321 protein residues (Figure 15).


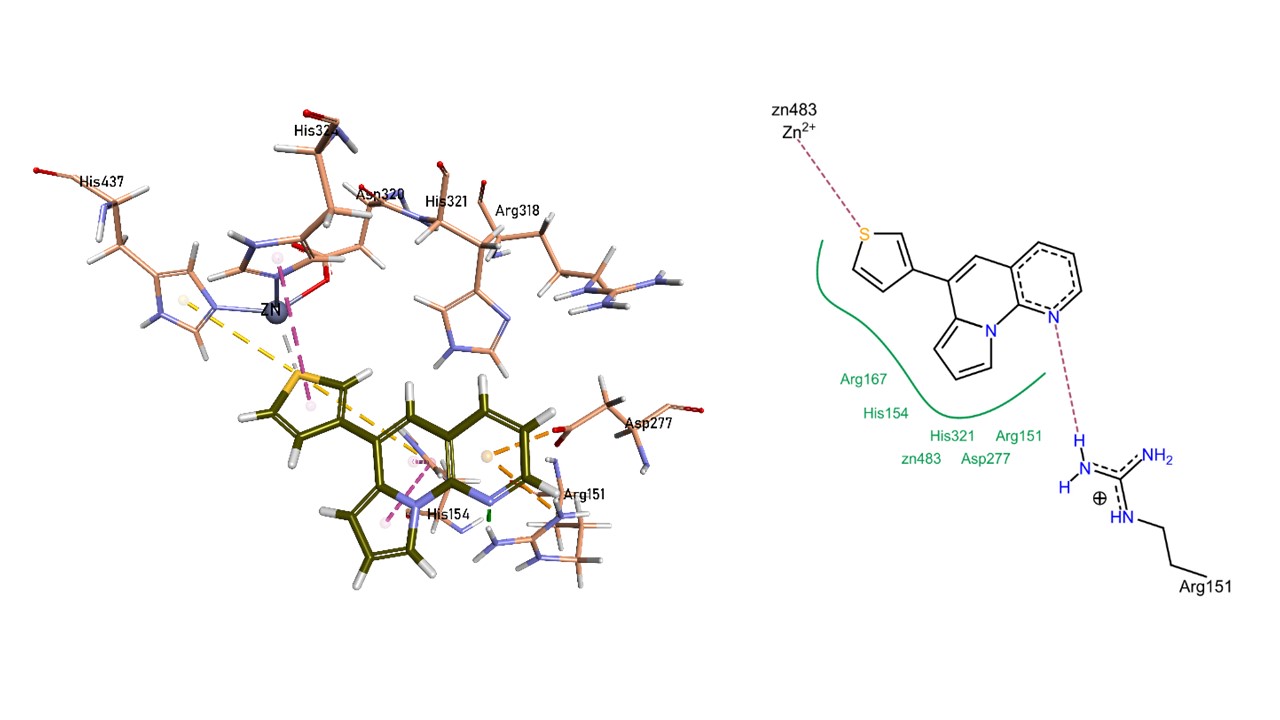


**Supplementary Figure 15** Compound **1k** interactions inside TNAP active pocket

The nitrogen heteroatom of pyrrolo-naphthyridine ring of 1l formed a metallic bond with Zn^2+^ metal and showed π-cationic interaction with Asp320 while π-anionic linkage with Arg167 residues. The cyclohexyl residue of **1l** compound exhibited π-σ linkage with His321residue. The compound 1l also exhibited van der Waals interactions with His154, Arg167, and His324

residues of the protein (Figure 16).


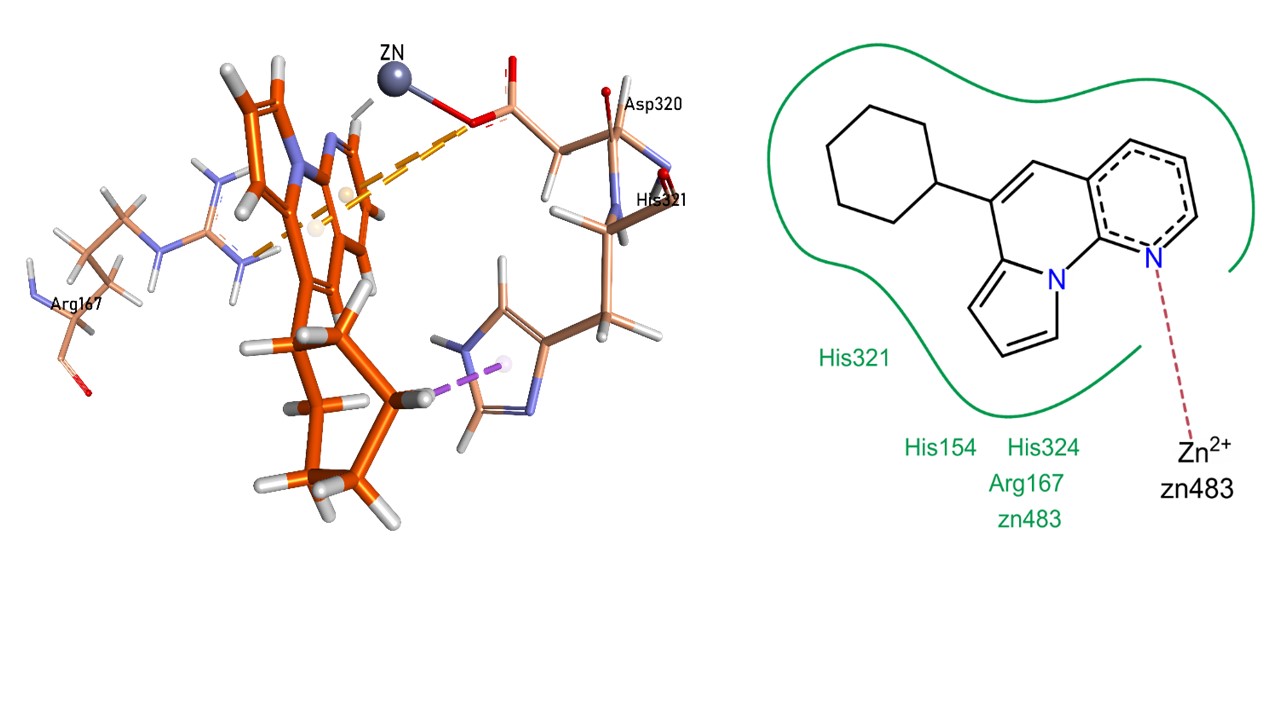


**Supplementary Figure 16** Compound **1l** interactions inside TNAP active pocket
